# Effectiveness of Self-Adhesive Resin Luting Cement in CAD-CAM Blocks—A Systematic Review and Meta-Analysis

**DOI:** 10.3390/ma16082996

**Published:** 2023-04-10

**Authors:** Maria João Calheiros-Lobo, Tatiana Vieira, Ricardo Carbas, Lucas F. M. da Silva, Teresa Pinho

**Affiliations:** 1 UNIPRO—Oral Pathology and Rehabilitation Research Unit, University Institute of Health Sciences (IUCS), Cooperativa de Ensino Superior Politécnico e Universitário (CESPU), Rua Central de Gandra 1317, 4585-116 Gandra, Portugal; 2 Conservative Dentistry, Department of Dental Sciences, University Institute of Health Sciences (IUCS), Cooperativa de Ensino Superior Politécnico e Universitário (CESPU), Rua Central de Gandra 1317, 4585-116 Gandra, Portugal; 3 Department of Mechanical Engineering, Faculty of Engineering, University of Porto, 4200-465 Porto, Portugal; 4 INEGI—Institute of Science and Innovation in Mechanical and Industrial Engineering, University of Porto, 4200-465 Porto, Portugal; 5 IBMC—Instituto Biologia Molecular e Celular, i3S—Instituto de Inovação e Investigação em Saúde, Institute for Molecular and Cell Biology (IBMC), Institute of Innovation and Investigation in Health (i3S), University of Porto, 4200-135 Porto, Portugal

**Keywords:** dental, tooth, self-adhesive, luting, cement, CAD-CAM, monolithic ceramics, blocks

## Abstract

Self-adhesive resin cements (SARCs) are used because of their mechanical properties, ease of cementation protocols, and lack of requirements for acid conditioning or adhesive systems. SARCs are generally dual-cured, photoactivated, and self-cured, with a slight increase in acidic pH, allowing self-adhesiveness and increasing resistance to hydrolysis. This systematic review assessed the adhesive strength of SARC systems luted to different substrates and computer-aided design and manufacturing (CAD/CAM) ceramic blocks. The PubMed/MedLine and Science Direct databases were searched using the Boolean formula [((dental or tooth) AND (self-adhesive) AND (luting or cement) AND CAD-CAM) NOT (endodontics or implants)]. Of the 199 articles obtained, 31 were selected for the quality assessment. Lava Ultimate (resin matrix filled with nanoceramic) and Vita Enamic (polymer-infiltrated ceramic) blocks were the most tested. Rely X Unicem 2 was the most tested resin cement, followed by Rely X Unicem > Ultimate > U200, and μTBS was the test most used. The meta-analysis confirmed the substrate-dependent adhesive strength of SARCs, with significant differences between them and between SARCs and conventional resin-based adhesive cement (α < 0.05). SARCs are promising. However, one must be aware of the differences in the adhesive strengths. An appropriate combination of materials must be considered to improve the durability and stability of restorations.

## 1. Introduction

CAD-CAM technology in dental medicine is developing, allowing protocol standardization and a predictable quality of dental restorations while reducing the production price [1,2], aiming to deliver materials at their highest quality [3], and enhancing the outgrowth of highly esthetic and functional restorative materials [4,5,6]. This technology has boosted impression and casting procedures [6,7,8,9], supplying easier and quicker indirect restorations, frequently without the requirement for provisional restorations or dental laboratories, allowing single-visit [4,8,9] inlays, onlays, veneers, or even full-contour crowns fabricated with several alternative materials with high survival rates [10,11,12]. Candidate materials may incorporate lithium disilicate glass-ceramic, leucite-reinforced glass-ceramic, feldspathic ceramic, zirconia, resin-matrix composites, polymer-infiltrated ceramic, or titanium [1,13]. Rehabilitation with CAD-CAM materials is becoming a standard dental technique due to high-tech digital technology based on image-capturing scanner devices, software, and integrated CAD-CAM systems [13,14].

Adhesive strength, or adhesive efficacy, refers to the ability of an adhesive to bond two surfaces together and resist separation. It measures the force required to pull the two surfaces apart once they have been joined by the adhesive. It depends on various factors, such as the type of adhesive, the nature of the surfaces being bonded, the conditions under which the adhesive is applied, and the time allowed for the adhesive to cure or dry. Luting cement adhesive strength is the ability of dental cement to bond to tooth structure or other dental materials effectively [15,16]. For each type of material, a previous treatment of the surface to be adhered to is required before applying the luting cement [13,17,18]. Conventionally, for resin-based materials (Cerasmart, Estelite, HZR-CAD HR2, Lava Ultimate, Katana Avencia, Paradigm, Shofu Block HC) and polymer infiltrated ceramic (such as Vita Enamic), aluminum oxide sandblasting (SB) or etching with hydrofluoric acid (HF), both complemented by the application of silane coupling agent, is recommended [17]. For all glass ceramics, etching with hydrofluoric acid complemented by silane is the standard surface treatment. However, for feldspathic- and leucite-reinforced ceramics (IPS Empress CAD, IPS. e max CAD, IPS. e max Press, Vita Mark II), HF 5% between 30 and 120 s is recommended. At the same time, for lithium disilicate glass-ceramic (Celtra Duo, Vita Suprinity), it is not wise to use HF concentrations greater than 4.9% for 20 s [19]. Materials that contain methyl methacrylate (MMA) improve the bonding of CAD/CAM poly(methyl methacrylate) (PMMA) resin materials (artBloc Temp) [17].

When adhering CAD-CAM ceramic to tooth substrates, luting cement is crucial for clinical success and restoration longevity, and adhesive luting is more favorable than non-adhesive luting, except in the case of zirconia [8,20,21]. Adhesive luting cement is categorized according to the adhesion strategy as a conventional multi-step resin composite cement combined with an etch-and-rinse or self-adhesive system, and as self-adhesive resin cement (SARC) [22,23].

Introduced at the beginning of the 21st century as a revolutionary cement with a time-saving clinical protocol, SARC was designed to be an easier-to-handle cement [24]. In the SARC protocol, surface treatment of the joint substrates is not required [25,26] because it allows bonding to an unconditioned tooth surface, without pretreatment with an acid or adhesive, theoretically with a similar adhesive strength as that of the established conventional multi-step resin cement [27]. However, for better adhesion, mild acids can be used to remove or modify the smear layer [28]. The adhesive strength was reported to be lower in systems where the smear layer was modified rather than removed [27]. In addition, air polishing devices (sandblasting), by increasing the roughness of hard dental tissues and restorative materials, have been reported to increase the adhesive strength of an SARC [9].

Unlike the first generation of SARCs that demand surface treatment by sandblasting and silanization, the silane-containing SARCs, recently released on the market, do not need the silanization step [25]. The chemical composition of these SARCs is based on methacrylate monomers modified by carboxylic or phosphoric acid groups, simultaneously demineralizing and infiltrating dentin and enamel without the need for separate etching and bonding steps, forming micromechanical interlocking and chemical bonding by interaction with the calcium ions of the tooth substrate [23]. After paste mixing, the phosphoric acid groups react with the hard tissue of the tooth and basic fillers in the luting material (cement reaction) to form a bond. In parallel with the cement reaction, the polymerization of methacrylate monomers is initiated (radical polymerization). Meanwhile, the acid groups are neutralized, turning the material’s behavior from hydrophilic to hydrophobic [24,26,29].

Despite being more straightforward, professionals must know that problems can occur during cementation with SARC. Lack of polymerization efficiency, with the potential release of unreacted cytotoxic and genotoxic monomers [30], induces expansion of the cement layer, with polymerization shrinkage strain and high stresses caused by hygroscopic expansion, with possible crack formation and restoration failure [26,28]. An evenly distributed cement layer with low internal gap values is essential for correct seating and better mechanical properties, but also for a low-space volume of the cement and porosities inside the luting agent [5]. Factors such as the cement mixing method or the particle size might amplify the formation of porosities [5]. Furthermore, differences in humidity, pH, and oral cavity temperature cause changes in dental materials [31]. SARCs exhibit good biocompatibility and marginal integrity, low microleakage [6], mechanical quality, and esthetic properties, being the most commonly used cements for the bonding of a restoration [30]. The adhesive strength of CAD-CAM ceramics to tooth substrates also depends on the type of ceramic, resin-matrix cement, the functional monomer used, and patient-related factors such as dentin thickness, occlusal loading, dental age, and proper oral hygiene [27].

Considering the existence of different adhesive strategies and that SARCs do not require additional steps for the adhesion of CAD-CAM restorations, it is necessary to clarify the adhesive strength of SARCs when cementing CAD-CAM ceramic blocks to tooth substrates. In parallel, it is also pertinent to assess the adhesive strength of each SARC when cementing different CAD-CAM ceramic blocks and compare their adhesive strength with conventional multi-step resin cements.

The first null hypothesis was that no differences exist in the adhesive strength between the self-adhesive resin-matrix cement systems used to lute CAD-CAM ceramic blocks. The second null hypothesis was that no differences exist between the self-adhesive resin-matrix cement and conventional resin-matrix cement used for luting CAD-CAM ceramic blocks.

## 2. Materials and Methods

The review followed the preferred reporting items for systematic reviews and meta-analysis (PRISMA) 2020 recommendations [32]. The population, intervention, comparison, and outcome (PICO) question was: “Are the self-adhesive resin-matrix cements efficient in luting CAD-CAM blocks?” The CAD-CAM blocks constituted the population. The intervention was defined as the cementation of blocks to dental and non-dental substrates. A comparison was made between each self-adhesive luting cement to determine intra- and interstudy differences in mechanical performance and between them and conventional resin-matrix luting cement. The adhesive strength was defined as the outcome.

### 2.1. Databases and Search Strategy

Bibliographic research was carried out in MedLine/PubMed and Science Direct databases with the keywords conjugated in the Boolean search formula: (“dental” [All Fields] OR “tooth” [MeSH Terms]) AND ((“self-adhesive” [All Fields]) AND (“luting” [All Fields] or “cement” [All Fields])) AND “CAD-CAM” [All Fields] NOT (“endodontics” [MeSH Terms] OR “implants” [All Fields]) and in Science Direct the keywords combined in the formula (“dental” or “tooth”) AND (”self-adhesive”) AND (“luting” or “cement”)) AND “CAD-CAM”) NOT (“endodontics” or “implants”), from 1 January 2012 to 31 July 2022, and again revised on 10 January 2023, for possible new entries.

### 2.2. Inclusion and Exclusion Criteria

Inclusion criteria were English language, accessible full-text research articles published in the last ten years, evaluation of adhesion strength between resin cement and dental and non-dental substrates, studies assessing microshear bond strength (µSBS), macroshear bond strength (SBS), microtensile bond strength (µTBS), and macrotensile bond strength (TBS) tests, and marginal parameter evaluation.

The exclusion criteria were non-CAD-CAM ceramic blocks, absence of bonding strength evaluation, data not presented in MPa or without a normal distribution, clinical trials, case reports, case series, pilot studies, encyclopedia articles, and articles published before 2012.

Preliminary removal of duplicate articles was performed using a citation manager (EndNote X9 Windows; Clarivate). Articles were then filtered by title, abstract, and complete reading in agreement with the PRISMA Statement.

Two investigators (M.J.C.L. and T.L.V.) independently selected each pertinent article for a detailed reading. A third investigator (T.P.) resolved any disagreements.

Additional research was conducted manually, pairing each word with the words self-adhesive and universal adhesives to identify relevant literature reviews, systematic reviews related to the subject, or other studies indirectly related to the topic to allow comparisons or enrich the introduction and discussion sections. 

### 2.3. Quality Assessment Protocol

The selected articles were included in this systematic review and subjected to quality assessment to determine the risk of bias (BIAS), which was calculated according to the following criteria: random distribution of the specimen, blind sampling by the operator, single operator, standardization of the specimen, control group, fractographic analysis, respect for the manufacturer’s instructions, compliance with international standards (ISO), sample size calculation, and statistical analysis quality.

The study’s publication date and the publication’s quotation by date in the SRJ score (Q_1_–Q_4_) were also analyzed. Qualitative analysis of the risk of bias assessment was performed by individually scoring the ten selected parameters using the following criteria: (0) clearly mentioned, (1) present but not accurately mentioned, and (2) not mentioned. Global scoring was categorized as low (0–4), medium (5–12), high (13–17), or very high (18–20) risk of bias. 

### 2.4. Data Extraction Workflow

Data extraction was performed, and the data were condensed into tables according to the item’s author, year of publication, CAD-CAM material, sample size, pairing (luted substrate), type of test performed, surface treatment, coupling agent used, adhesive system used, and luting cement tested. The mean and standard deviation of the bond strength were recorded in MPa, and the values for marginal adaptation were registered for statistical treatment.

### 2.5. Meta-Analysis

A meta-analysis of the adhesive strategies for each luting cement brand was conducted using a software program (Stata v17.0; StataCorp, Lakeway, TX, USA). Subgroup analyses assessed the different types of surface treatment methods, adhesive joint substrates, and mechanical tests. For all studies that evaluated more than one type of CAD-CAM block or more than one surface treatment method, each type of material or treatment method was considered independently.

Statistical heterogeneity was determined using the I^2^ test (α = 0.05). A meta-analysis was conducted by the author and the CAD-CAM block to determine intrastudy heterogeneity and protocol splitting by efficiency after calculating the difference between means and effect size (α = 0.05; 95% CI; Z-value 1.96) (Table S1). Funnel and Galbraith’s plots assessed publication bias and heterogeneity (random-effects model; α = 0.01; 99.9% CI; Z-value 2.58).

## 3. Results

### 3.1. General Aspects

**Figure 1:**
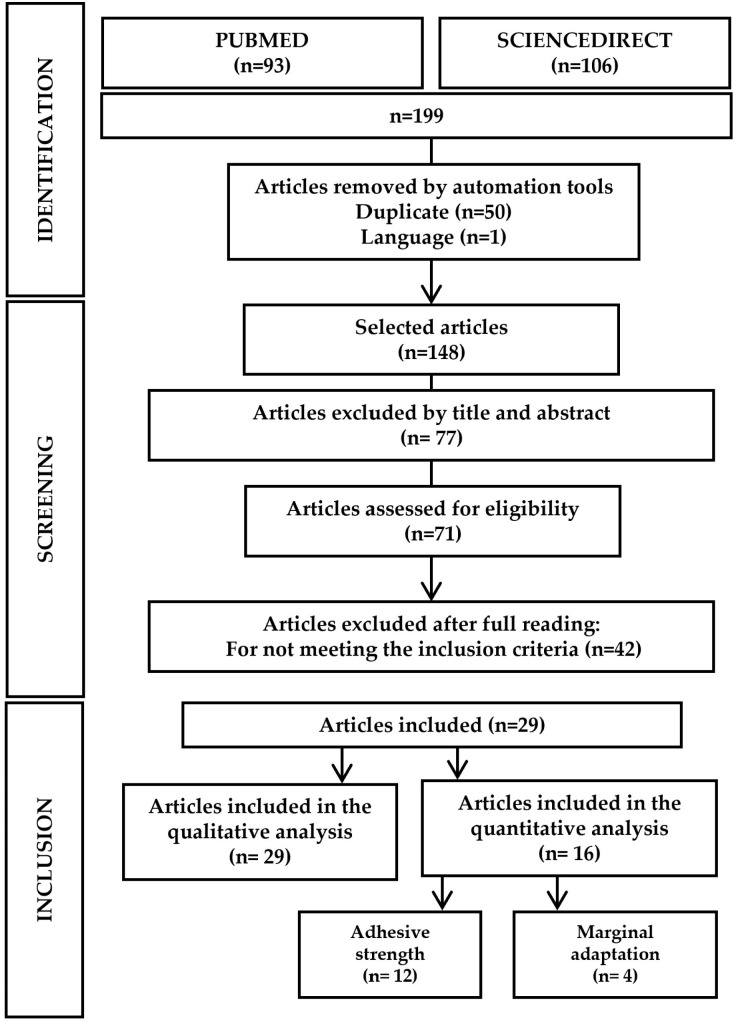
Flow diagram of study selection according to the PRISMA statement.

The search retrieved 199 articles [Medline/PubMed (93) and ScienceDirect (106)]. One article was immediately excluded based on language, and 50 were duplicate publications. Seventy-seven articles were removed by reading the titles and abstracts, and 42 were removed after complete reading. The remaining 29 articles [1,2,3,4,5,6,7,8,9,10,14,22,25,26,27,28,31,33,34,35,36,37,38,39,40,41,42,43,44] were selected for the quality analysis. Manual research also retrieved three studies [30,45,46], three randomized clinical trials [24,47,48], six reviews [15,23,29,49,50,51], and three meta-analyses [11,12,20], which were used to broaden the introduction and discussion sessions. The selection process agreed with the PRISMA Statement, as shown in Figure 1.

### 3.2. BIAS Risk Assessment

Qualitative analysis for risk of bias assessment (Table 1) revealed one low-risk [34] (3.45%) and 30 medium-risk of bias (96.55%) articles. Transversal factors for lower scores were the absence of operator blindness (referred to in two articles [3,34] (6.9%)) and no reference to a single operator (referred to in five studies [6,9,33,34] (13.79%)).

The description of specimen randomization and the control group were frequently inadequately described or lacking. The journal rankings are Q1 (62.07%), Q2 (34.48%), and Q3 (3.45%).

### 3.3. Descriptive Data

Data extraction recovered the information summarized in Table 2 and Table 3. A synopsis of the CAD-CAM materials evaluated in the studies by type and physical properties is presented in Table 4. Lava Ultimate and Vita Enamic blocks were the most tested CAD-CAM blocks. Rely X Ultimate 2 was the most widely used resin cement, followed by Rely X Unicem, Rely X Ultimate, and Rely X U200, and μTBS was the most used test.

### 3.4. Meta-Analysis

For quantitative analysis, 12 studies were sub-grouped to evaluate mechanical performance [1,2,6,7,8,9,10,25,27,35,37,42] and four [3,4,5,26] to evaluate marginal parameters. Five articles initially thought to be included were rejected for the meta-analysis because they provided no quantitative results, making inclusion impossible for mechanical [14,38,40] or marginal assessment [22,28]. Table 5 lists the blocks found in the studies that evaluated the mechanical performance and the relative number of tests available.

**Figure 2:**
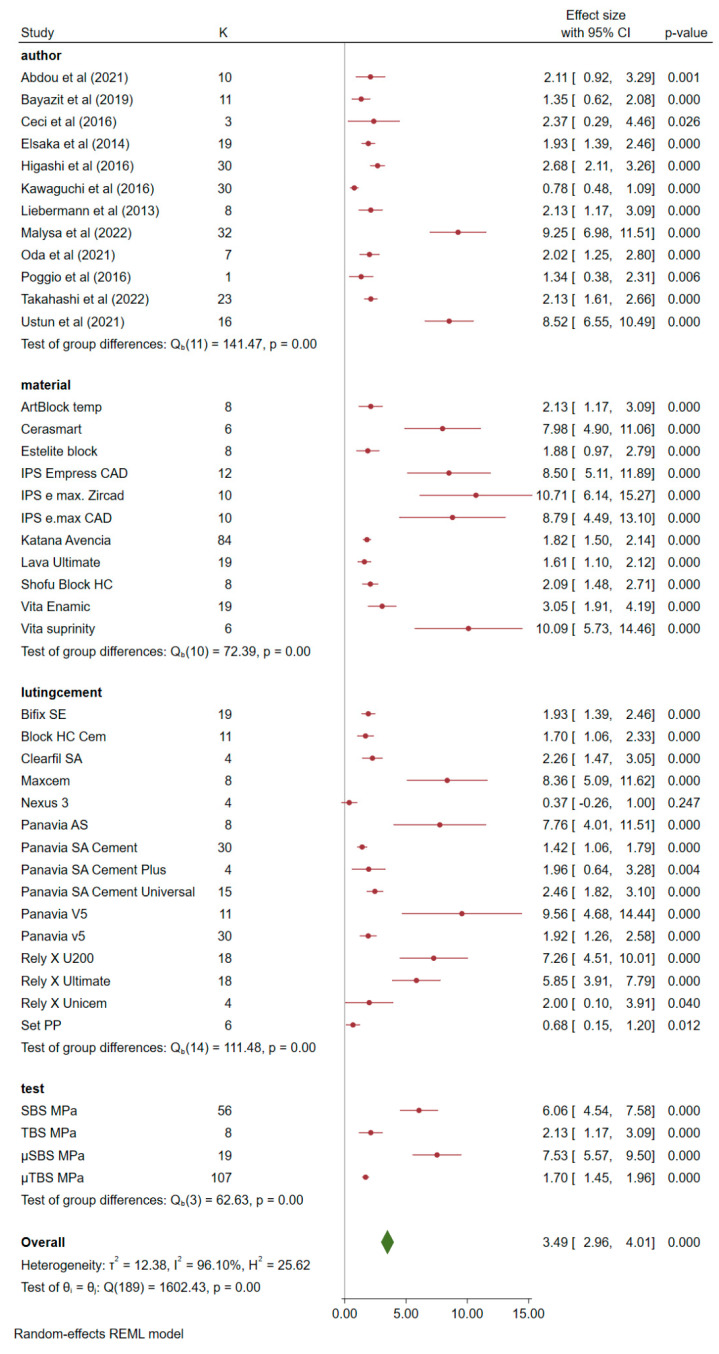
Forest plot summarizing the effect size of the author, CAD-CAM block, luting cement, and mechanical tests with data obtained from the included studies [1,2,6,7,8,9,10,25,27,35,37,42].

The meta-analysis combining the selected 12 articles based on the difference between means and the effect size (*p* = 0.05; 95% CI; Z-value 1.9599) for mechanical performance is shown in Figure 2.

**Figure 3:**
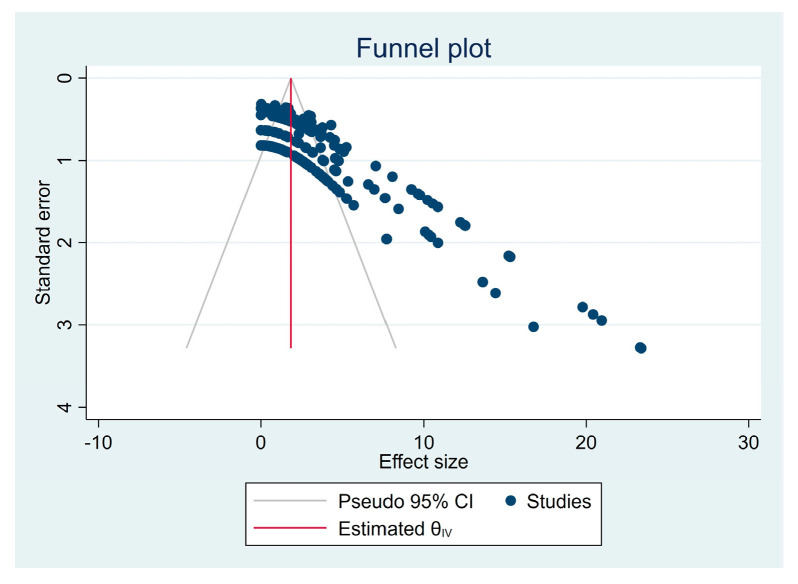
Funnel plot of publication bias of all selected publications, filtered by the joint substrate and mechanical tests.

**Figure 4:**
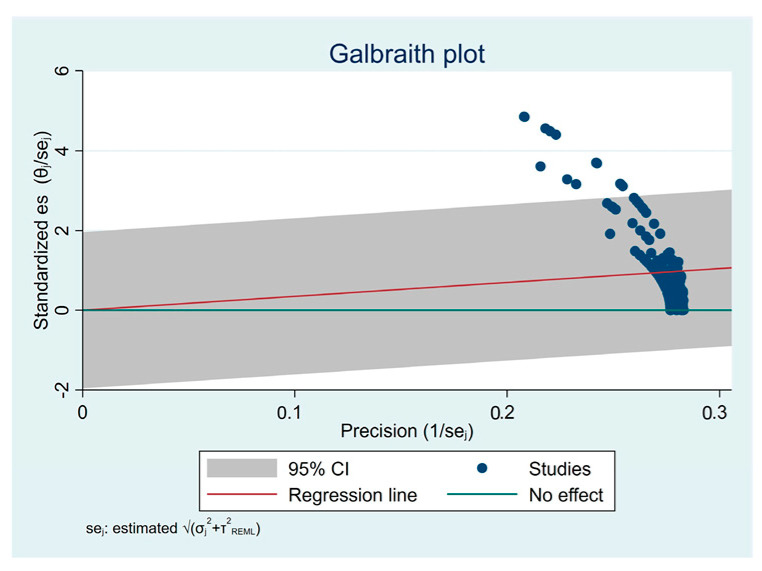
Heterogeneity assessment of effect sizes.

**Figure 5:**
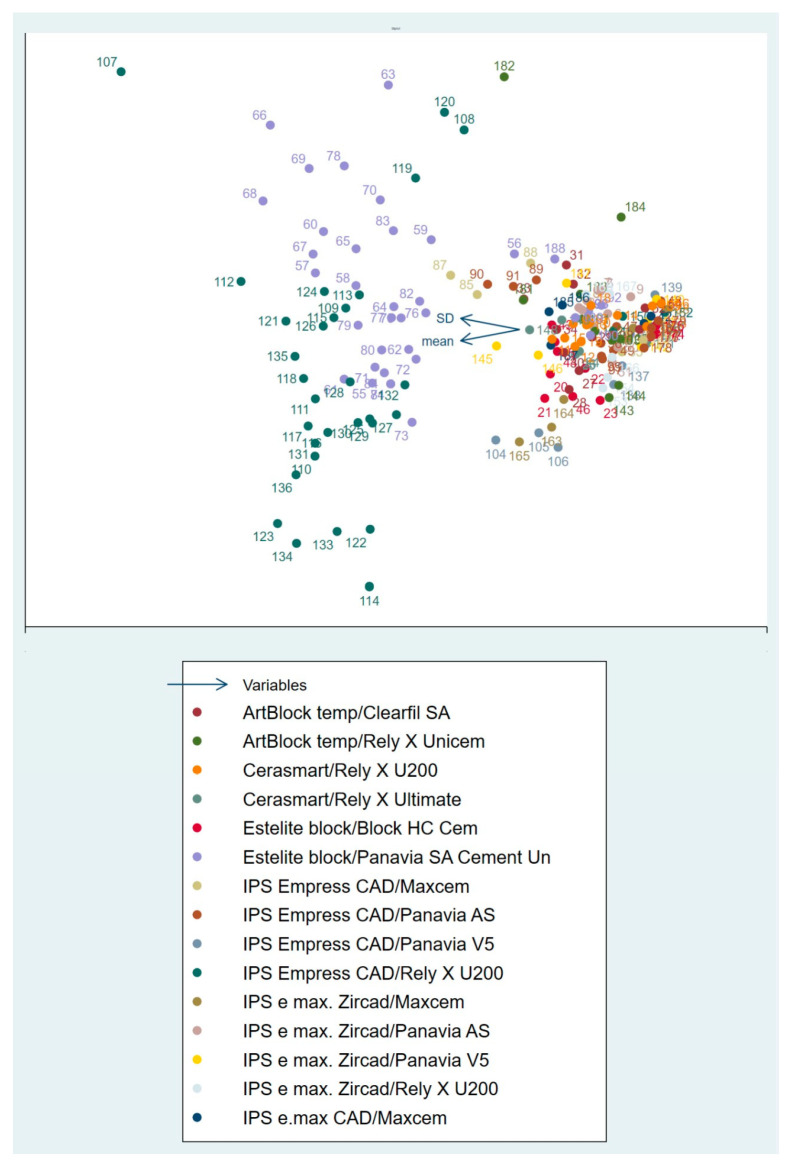
Biplot graphs of mean and standard deviation (SD).

The assessment of publication bias and heterogeneity for these subgroups of articles is shown in Figure 3 and Figure 4, respectively. Heterogeneity is expected when assessing studies with different tests and substrates. Even so, it is essential to analyze this heterogeneity, as it is entirely different to find a total dispersion of studies or to find a tendency towards aggregation, as is the case. Funnel plot asymmetry suggests an overestimation of the intervention effect, probably induced by the disparity between samples, with some possible bias. The Galbraith plot suggests some heterogeneity among the effect sizes, as although most of the studies were within the 95% CI region, several were outside. All studies had high precision (toward the right of the X-axis). Globally, the studies were above the green line, with the red line sloping upward, suggesting favorable testing protocols compared to the control protocol. The biplot graph in Figure 5 displays the means and standard deviations (SD) of some tested material–luting cement pairs and reveals heterogeneous mechanical performance among the tested protocols. The graph suggests a similar behavior for most pairs of adhered substrates but also some performance disparities.

**Figure 6:**
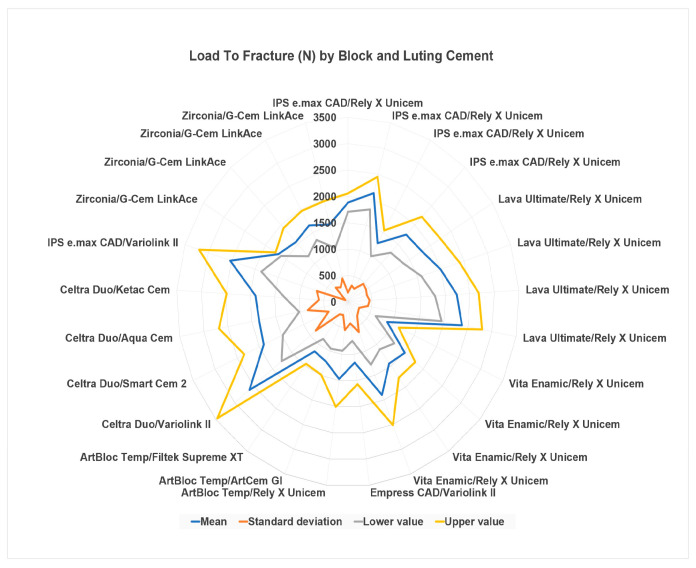
Radar graphs with load-to-fracture by CAD-CAM block and luting cement. [1,2,6,7,8,9,10,25,27,35,37,42].

Figure 6 shows that the Variolink II cement provides resistance to Celtra DUO blocks and IPS emax CAD. The latter is also resistant when cemented with Rely X Unicem, a cement proven to exhibit excellent and universal performance.

**Figure 7:**
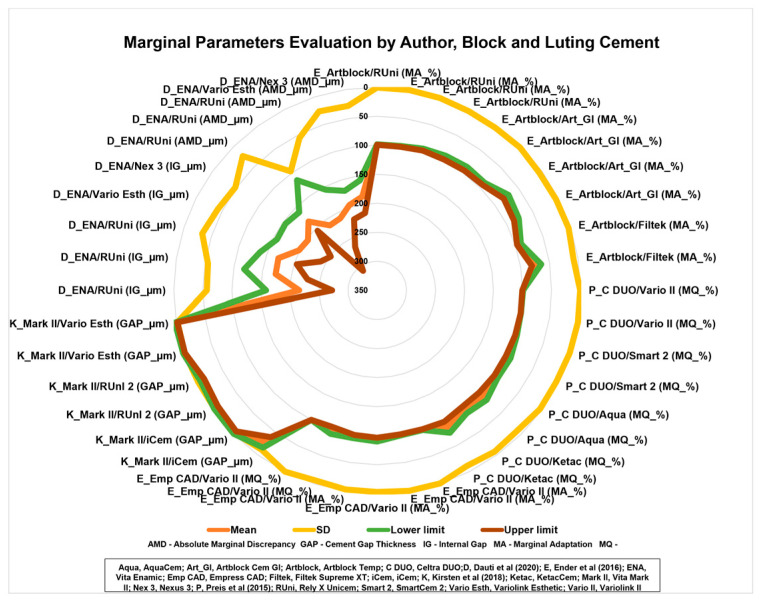
Radar graphs with marginal parameter evaluation by author, CAD-CAM block, and luting cement [3,4,5,26].

Figure 7 shows that the Vita Enamic block has an irregular behavior for the marginal parameters evaluated, regardless of the cement used. Concerning the marginal gap, the Vita Mark II (feldspathic ceramic) has an excellent marginal fit.

## 4. Discussion

This review assessed whether self-adhesive resin-matrix composite cement (SARC) is adequate for the luting cementation of CAD-CAM ceramic blocks and which is the best luting cement adhesive protocol for each block. Based on the existing data (*p* < 0.05), it was accepted that self-adhesive resin-matrix cement systems are effective in cementing CAD-CAM blocks on different substrates and rejected the hypothesis that self-adhesive resin-matrix cement performs better than conventional resin-matrix cement. Moreover, it was not possible to establish a luting cement that suits a particular CAD-CAM block, or if there is a better SARC adequate for all situations, which agrees with a recent publication for luting protocols [11].

Before a detailed discussion of the results of the available studies, general considerations must be made. When evaluating laboratory studies, one must always consider that their ultimate purpose should be to find solutions that can be implemented in a clinical environment to improve the quality of restorative options. In addition, the adhesive cementation of a CAD-CAM ceramic restoration to dental structures depends on a complex adhesive joint. This joint is formed by two interfaces: one between the dental structures (enamel and/or dentin) and the luting cement, and the other between the luting cement and the CAD-CAM ceramic. This last aspect has led to research focusing on adhered restorations as a whole, on the cement–tooth interface, or on the cement–restoration interface. It should also be mentioned that using bovine teeth for laboratory tests is a common practice that overcomes some ethical constraints of using human teeth. These teeth are considered credible substitutes, with a mechanical and adhesive behavior similar to human teeth [52,53]. Finally, to overcome the fact that the substrates used in the studies, as well as the protocols tested, were frequently different, the adhesive strength was compared only between blocks studied in at least two studies and within the same study each adhesive protocol was compared used with different CAD-CAM blocks.

Several studies have been conducted on SARCs. SARCs exhibited different adhesive strengths depending on which CAD-CAM block was evaluated, how the surface was treated, and which luting cement was used. Thus, many criteria must be considered for the luting success of the CAD-CAM blocks.

Adhesive cementation with SARC is less technique-sensitive and time-consuming than conventional methods because it bonds to an unconditioned tooth surface without the need for pretreatment with an acid or adhesive, allowing placement of the restoration in a single step. However, several strategies to treat the substrate surface before applying self-adhesive resin cement have been developed to improve bond strength.

### 4.1. Surface Treatment

The most frequently used treatment in the selected studies was sandblasting with 50 μm aluminum oxide particles (Al_2_O_3_). For resin-matrix ceramic, surface treatment is the most critical factor affecting the bond strength between the resin cement and the CAD-CAM material, followed by the type of resin-matrix ceramic and the resin cement, respectively [8]. Sandblasting has been proposed as the preferred pretreatment for CAD-CAM hybrid ceramics with high ceramic content, such as Vita Enamic [8]. In contrast, pre-treatment with hydrofluoric acid (HF) is recommended for CAD-CAM resin nanoceramics reinforced with nanoparticles, such as Lava Ultimate. Nevertheless, it was found that in hybrid ceramics, such as Vita Enamic, surface treatment with HF and a silane coupling agent showed higher bond strength values than sandblasting or HF alone. Vita Enamic coupled with Bifix (SARC) appears more hydrolytically stable and durable than Lava Ultimate coupled with the same SARC [1,35]. Recently, it was advocated that sandblasting or HF followed by a universal adhesive could also be used with effectiveness as pre-treatment [15].

Other studies [14,38] found that the priming or sandblasting of the CAD-CAM composite and ceramic blocks significantly increased the bond strength of SARCs compared to non-treated controls. In addition, bond strengths obtained by 9% HF etching and priming were comparable to those obtained by sandblasting and priming [38]. Other surface treatments were investigated in different studies, such as polyacrylic acid, with no significant difference in the interfacial adaptation of resin nanoceramic inlays [28]. Additionally, surface treatment with plasma of an organic modified polymer infiltrated network (PMMA) block did not increase the adhesion to SARC despite increased surface energy, with no impact on surface roughness and a negative impact on the bonding with dental resin-matrix materials [2]. Furthermore, pre-treatment with glycine did not significantly change the bond strength in the various luting protocols tested. Still, it increased the bond strength of self-adhesive resin cement, so it needs further investigation [9]. Studies concerning ultrasonic and acid cleaning after sandblasting suggest that as long as the restorations are sandblasted after the try-in procedure in a clinical setting, there is no need for ultrasonic and acid cleaning after sandblasting to improve the microtensile bond strength [10].

Disparities were described in the optimal surface treatment and resin cement selection for Vita Enamic and Lava Ultimate resin-matrix ceramic blocks [40]. For Lava Ultimate (resinous matrix composite densely packed with silica and zirconia particles), sandblasting pretreatment was proposed, but hydrofluoric acid etching significantly positively affected bond strength. In terms of resin cement, the self-adhesive material (Panavia SA Cement) outperformed the conventional resin cement (Clearfil Esthetic Cement) in terms of bond strength to Lava Ultimate [40]. Today, Lava Ultimate is still indicated for inlays, onlays, and veneers, but the manufacturer has removed the crown indication since June 2015 because of the higher rates of premature debonding. Recently, a meta-analysis [11] revealed as a better protocol for Lava Ultimate, sandblasting with 50 µm Al_2_O_3_ and an SARC (G-Cem LinkForce) + Universal Primer (G-Multi Primer), and as the worst protocol, the use of no sandblasting and an SARC (RelyX Unicem 2) used alone. In contrast, the surface treatment had little effect on the bonding to Vita Enamic (a ceramic structure infiltrated with resin). The manufacturer recommends silane as the best surface treatment, alone or after HF. However, the self-adhesive resin cement demonstrated a lower overall bond strength within the same surface treatment group than conventional resin cement [35]. This variance in results can be explained by using different methodologies and materials and the lack of a separate adhesive layer in self-adhesive resin cement. Even though some results are contradictory, most studies recommend HF and silane as surface treatments for Vita Enamic or a universal primer [11,15].

Concerning the fabrication of monolithic zirconia crowns with reduced crown thickness to a lower limit of 0.5 mm, it was described that regardless of the cement type, the crown still had sufficient strength to withstand occlusal loads, with less invasiveness of the preparation and tooth tissue preservation [39,41]. Furthermore, adequate retention and resistance designs heightened the zirconia coping retention compared to copings cemented on teeth lacking these forms. Interestingly, upon failure, the cement mainly remained on the tooth if an adhesive resin cement was coupled with a bonding system. In contrast, the cement remained mainly on the coping with self-adhesive resin cement [33], reflecting adhesive failure. When comparing the bonding strength between a felspathic ceramic, a disilicate ceramic, and a zirconia ceramic bonded with three different SARCs and a conventional multi-step resin cement, the zirconia ceramic had the lowest bond strength among the tested ceramics, regardless of the tested cement [37], highlighting the possibility of using another strategy for this material whenever esthetic issues are absent [15,50].

### 4.2. Interaction between Substrates

Since SARC reacts superficially with mineralized tissues, this self-adhesive resin cement does not form a strong dentin hybrid layer or resin tags [25]. Resin coating with a hydrophobic resin may be suggested, as it creates a layer with a low modulus of elasticity that acts as a stress breaker or shock absorber, resulting in higher bond strengths with the resin-coated groups, strengthening the dentin interface, thus leading to better adhesive performance, regardless of the resin cement and its curing mode [7,25,50].

The dual-curing mode exhibited a higher bond strength than the self-curing mode. The slow-curing process in the self-curing mode allows water to be absorbed from the dentinal tubules by osmosis. Therefore, a resin coating plays a role in suppressing water penetration through the adhesive layer, especially in the self-curing mode [23,25,50]. Furthermore, single-visit treatment results in a higher bond strength between resin cement, dentin, and CAD-CAM blocks than multiple-visit treatments, even with resin coating [7,23].

In general, self-adhesive resin cement is inherently a self-etching material during the initial stages of its chemical reaction. After mixing, its low pH and early high hydrophilicity result in good tooth structure wetting and promote surface demineralization, similar to self-etching adhesives [23,50]. As the reaction progresses, cement acidity is gradually neutralized by the reaction with the tooth substrate apatite and the metal oxides contained in the basic and acid-soluble inorganic fillers. Cement becomes more hydrophobic as chemical reactions in situ consume hydrophilic and acidic monomers. This is highly desirable in a fully cured resin to minimize water sorption, hygroscopic expansion, and hydrolytic degradation [26].

Self-adhesive resin cement with a lower pH-neutralizing capacity has higher residual hydrophilicity and higher hygroscopic expansion [45]. Water sorption and significant hygroscopic expansion stresses can result from the residual hydrophilicity during and after the setting reaction. Whenever a self-adhesive resin cement is a clinical option, cement with a strong neutralization reaction is recommended, resulting in lower hygroscopic expansion strain [45]. Cracks can be attributed to the hygroscopic expansion stress of the build-up and luting material, and it is possible that the storage of specimens in distilled water increases the rate of water uptake, resulting in higher hygroscopic expansion stresses [26].

Incorporating acidic monomers with hydrogen bonding sites, such as hydroxyl, phosphate, or carboxyl groups, contributes to the natural hydrophilicity of SARCs compared with conventional resin cement. SARCs with poor pH neutralization and high hygroscopic expansion stress can cause fractures in feldspathic ceramic crowns. This phenomenon can be increased by pre-damaging during CAD-CAM processing [26]. For this, in clinical use, in conjunction with CAD-CAM crowns, SARCs with increased pH neutralization behavior and low hygroscopic expansion stress are preferred [15,22].

### 4.3. Adhesive Strategy

Luting strategies fall into adhesive or non-adhesive strategies, but adhesive luting reinforces the mechanical properties of the CAD-CAM ceramic used as a restorative material, excepting zirconia polycrystals [20]. An adhesive luting strategy could be conventional multi-step or self-adhesive [50]. Self-adhesive resin cement aimed to reduce these conventional steps [8]. Conventional multi-step resin luting (with etch-and-rinse, self-etch adhesives, or priming) enables higher adhesive strength values of the bonding interfaces than the self-adhesive strategy alone [1,11,42,45], especially when a conventional resin cement is combined with a self-etch adhesive [6].

The clinical use of SARC results in less postoperative sensitivity than resin-modified glass ionomer cement and glass ionomer cement. However, the adhesive strength values of self-adhesive resin cement bonded to both enamel and dentine are lower than those of conventional multi-step resin cement [50].

This study found self-adhesive resin cement is not recommended for restorations with reduced retention and resistance, such as resin-bonded bridges and crowns with low heights. This is in line with the literature [50]. Similarly, veneers require a strong bond to the tooth structure to ensure their longevity and prevent discoloration; self-adhesive resin cement may not provide the necessary bond strength, especially in cases with weak enamel bonding [50]. In such cases, conventional or dual-cure resin cement may be more appropriate [45,50].

Assessment of the fatigue resistance of ultrathin CAD-CAM crowns cemented with SARC (Rely X Unicem 2) revealed the possibility of using resin nanoceramics and lithium disilicate to restore posterior teeth with regular or ultrathin crowns, even with relatively high loading requirements. However, SARCs should not be used for ultrathin crowns with feldspathic ceramic veneers. The immediate dentin sealing (IDS) technique should be used with preheated composite resin as a luting agent [36]. Poly(methyl methacrylate) (PMMA)-based CAD-CAM inlays, luted with self-adhesive resin cement, may be applied as long-term restorations in narrow cavities based on the findings of marginal adaption, fracture load, and fracture analysis [3].

Considering the material strength and chemical characteristics of Vita Suprinity ceramic restorations, both total-etch and self-adhesive systems may be recommended. However, the self-adhesive systems with a lower pH neutralizing capacity allow more hydrolysis and chemical degradation over time than a total-etch system [27,45]. Furthermore, self-adhesive rather than total-etch systems are appropriate for performing Vita Suprinity ceramic restorations in deep cavities with high postoperative sensitivity. It is possible to recommend cementing Vita Enamic and GC ceramic restorations with self-etch systems. Regardless of the cementation system, the thermal aging process significantly reduced the bond strength values of all ceramic materials [27].

Only three clinical studies were found during the manual search [24,47,48]. A prospective randomized clinical trial (RCT) testing the selective etching of enamel in the cementation of partial ceramic crowns with SARCs [24] with control at 12, 24, and 36 months found the potential to improve restoration survival rates in challenging clinical situations. Another RCT with control at 6, 12, and 18 months found no statistically significant difference in the survival rates, surface texture, secondary caries, anatomic form, color match, marginal discoloration, marginal integrity, interproximal contacts, and patient satisfaction between CAD/CAM-fabricated resin nanoceramic inlay/onlay restorations cemented with either a self-adhesive after selective enamel etching or a universal adhesive/resin cement system [47]. In contrast, an RCT using a split-mouth model, with evaluation after 39 months, found significant differences in luting adhesive strategies and stated that self-adhesive resin cements could not be recommended for luting partial ceramic, but instead, a luting procedure with a luting composite coupled with a universal adhesive yielded promising clinical results with or without the use of a selective enamel etching step [48].

Currently, SARCs are not recommended for luting partial ceramic crowns.

Regarding the most effective cementation protocol for bonding zirconia crowns to Ti-base CAD-CAM abutments in terms of abutment height, cement type, and surface treatment, it was found that conventional resin cement associated with self-etch adhesive displayed higher retention than self-adhesive cement and that high abutments presented higher retention pressures than short ones. Hierarchically, the results showed a direct correlation between Ti-base height, micro-mechanical and/or chemical pre-treatment, Ti-base surface blasting, and zirconia, and that tribochemical and silica coating increased the retention of zirconia crowns, followed by Ti-base surface blasting or tribochemical silica coating [43].

### 4.4. Coupling Agents

The association of a universal adhesive or primer with self-adhesive resin cement to attach to CAD-CAM composite blocks significantly increased the bond strength compared with the self-adhesive resin cement used alone for the same period. Still, surface treatment is a more important factor affecting the bond strength of resin cement to the resin-matrix ceramic than the specific cement used [8,38]. Recently, it was suggested that silane could be successfully adjoined to the hydrophobic paste of a self-adhesive composite cement, eliminating the need for a separate silanization step, thus simplifying the adhesive bonding process [44]. SARCs must be presented as two-part materials, usually in separate individual syringes or more popular dual-barrel syringe dispensers [45], with the last presentation being unfavorable for silane addition.

However, a study that evaluated the bond strength between nanoceramic materials and bovine dentin using various adhesive systems reported that conventional multistep resin cement (coupled with etch-and-rinse or self-etch adhesives) showed better shear strength values than SARCs. Moreover, association with self-etch adhesive resulted in the highest values of adhesion bonds, and adding silane to the surfaces of the resin matrix ceramics increased the shear bond strength [6].

The one-step self-etch adhesive differs from two-step self-etch adhesive. An extra hydrophobic bonding resin applied over the acidic primer for the two-step self-etch adhesives turns it into the gold standard for the self-etch strategy [23]. Nevertheless, most universal adhesives must be mixed with the respective dual-cure activator when used with self- or dual-cure composite materials, such as build-up materials and resin cement with aromatic tertiary amines in the initiator system [23].

When adhering to the tooth structure, selective enamel etching with phosphoric acid (PA) is recommended without etching the dentin, allowing potential chemical bonding between the functional monomer and dentin hydroxyapatite. Universal adhesives may also need extra solvent drying time to ensure the removal of the residual water in the interface [23].

Systematic reviews evaluating adhesion to zirconia have shown that using MDP-based self-adhesive cement yields more favorable results after physicochemical conditioning of the zirconia surface. Although water storage may affect the bond strength of resin cement to zirconia, no difference was found between the cements for a specific aged dataset. This may confirm that cement choice is less critical for zirconia-bond durability [45].

### 4.5. Dimensions of the Interface and Marginal Adaptation

Milled ceramic restorations cemented with self-adhesive resin cement result in a thinner cement line with the highest interface quality correlated with a thin cement interface [22]. Concerning hybrid ceramics (polymer-infiltrated ceramic network) crowns, the marginal and internal fits were not significantly affected by the different virtual spacer settings of 50 µm and 80 µm, and for those settings and three different resin luting materials (Rely X Unicem, Variolink Esthetic DC, Nexus 3) no significant influence was discovered in the marginal and internal fit [5]. In all investigations, porosities in the cement space on the periphery in contact with the outside environment were found. In the clinical setting, unprotected dentin can be contaminated through these voids by fluids, bacteria, and bacterial toxins, which could compromise the efficacy of the restoration [5]; a fact associated with plaque accumulation and a higher bleeding score around prostheses cemented with the resin cement [45] advises the removal of excess material at the restoration margins after brief light-activation (2–3 s from each side) in the case of light- or dual-cured cement [26].

As for the excess cement at the marginal adaptation, despite the cleaning process, similar quantities of undetected cement remnants were found around the esthetic margins of zirconia crown copings, regardless of the type of cement (conventional glass-ionomer or SARC). Cleaning procedures with clinically accessible instruments did not allow the complete removal of excess cement [34].

### 4.6. Toxicity and Aging

The in vitro cytotoxicity of an SARC used to cement a zirconia crown seems to be influenced by the inclination of the crown cusps, regardless of the curing time (20 s or 40 s). However, the cytotoxicity of a zirconia crown with a thickness of 1.0 mm conforms to ISO standards when the cusp inclination is less than 20 degrees but does not meet those standards when the cusp inclination of zirconia reached or exceeded 30 degrees [30]. In addition, the in vitro cytotoxicity of SARCs can be reduced by extending the light-curing period, which aligns with the literature for other restorative resin composites [29]. SARCs have different cytotoxic and apoptotic effects that increase with increased exposure time to non-converted monomers [46], drawing attention to the need for efficient polymerization and excess removal.

Among the studies found, the parameter of aging is not always considered or is not standardized, in line with the literature [11,20]. Generally, thermal aging reduces the bond strength values of all the interfaces studied, regardless of the cementation procedure. Still, resin cements are less prone to degradation in water than conventional acid-base cements and can maintain their properties for extended periods [1,11,20,27,37]. Clinicians should consider these variables and choose the most suitable cementation systems for each material [11,27].

Thermocycling affected the shear bond strength of self-adhesive, self-etching resin cements luted to human dentin and CAD-CAM ceramics, revealing that conventional resin cement (Panavia V5) demonstrated a significantly higher bonding strength than self-adhesive and self-etching cements, with significant differences in the bond strengths for the studied combinations. The most significant decreases in bond strength were observed for self-etching, self-adhesive cements when comparing samples that had not been thermocycled to those that had been artificially aged [37].

In addition, aging and deterioration often occur without visible catastrophic failures, particularly with high-strength ceramics [4]. Zirconia-reinforced lithium silicate ceramics exhibit high flexural strength and, at the same time, high translucency. Strong fracture forces, high resistance to aging, and good-to-adequate marginal adaptability have been observed, [4] indicating that no limitations should be anticipated for clinical use.

Glass ionomer cement, resin, and resin-modified self-adhesive luting materials are suitable for the cementation of molar crowns made of zirconia-reinforced lithium silicate ceramics.

## 5. Conclusions

SARCs perform well in mechanical tests but differ and do not necessarily produce similar results. Surface treatment of CAD-CAM ceramic restorations is mandatory before cementation, regardless of the SARC type. The effect of the surface treatment is material-dependent. For all types of ceramics, surface treatment or cement light-curing improved the adhesion compared with the SARC used alone and in the self-cured mode. Sandblasting is preferred for hybrid ceramics, while hydrofluoric acid is recommended for resin nanoceramics reinforced with nanoparticles and glass ceramics. A cement line with a reduced thickness correlates with a better interface quality. SARCs with increased pH neutralization behavior and low hygroscopic expansion stress are preferred for clinical use, and an extended light-curing time reduces the in vitro cytotoxicity of SARCs. Immediate dentin sealing improves the bond strength between dentin and CAD-CAM ceramic blocks. Single-visit treatments yield a higher bond strength than multiple-visit treatments. CAD-CAM zirconia crowns with an occlusal thickness of 0.5 mm, cemented with an SARC, withstand occlusal loads. Glass ionomer cements, resin, resin-modified glass ionomer cement, and self-adhesive luting materials are suitable for the cementation of molar zirconia-reinforced lithium silicate crowns, and the cement is less critical for bond durability than proper tooth preparation, cleaning, and drying before cementation. Dual-cured self-adhesive resin cements provide significantly higher early retention values than resin-modified glass ionomer materials. Each CAD-CAM material/luting composite association must be individually studied and evaluated to determine the optimal bonding protocol. There is an urgent need for randomized clinical trials or at least an extensive, well-documented series of clinical cases.

## Figures and Tables

**Table 1:** BIAS risk assessment and SJR scoring.

Study	Abdou et al. (2021) [7]	Albelasy et al. (2021) [31]	Ali et al. (2012) [33]	Augusti et al. (2020) [34]	Bayazit et al. (2019) [8]	Ceci et al. (2016) [9]	Dauti et al. (2020) [5]	Elsaka et al. (2014) [35]	Ender et al. (2016) [3]	Melo Freire et al. (2017) [22]	Han et al. (2020) [28]	Higashi et al. (2016) [1]	Kawaguchi et al. (2016) [10]	Kirsten et al. (2018) [26]	Liebermann et al. (2013) [2]	Magne et al. (2015) [36]	Malysa et al. (2022) [37]	Nagasawa et al. (2021) [38]	Nagasawa et al. (2022) [14]	Nakamura et al. (2016) [39]	Oda et al. (2021) [25]	Peumans et al. (2016) [40]	Poggio et al. (2016) [6]	Preis et al. (2015) [4]	Sorrentino et al. (2016) [42]	Takahashi et al. (2022) [43]	Ustun et al. (2021) [27]	Zahoui et al. (2020) [44]	Zhang et al. (2018) [30]
Specimen Randomization	1	1	0	1	0	0	0	0	0	0	0	0	0	0	1	1	1	1	1	0	0	0	0	1	0	1	1	1	1
Single Operator	2	2	0	0	2	0	2	2	2	2	2	2	2	2	2	2	2	2	2	2	2	2	0	2	2	2	2	2	2
Operator Blinded	2	2	2	0	2	2	2	2	0	2	2	2	2	2	2	2	2	2	2	2	2	2	2	2	2	2	2	2	2
Standardized Specimens	0	0	0	0	0	0	0	0	0	0	0	0	0	0	0	0	0	0	0	0	0	0	0	0	0	0	0	0	0
Control Group	2	2	2	2	0	2	2	0	0	2	0	0	0	2	0	2	0	0	0	2	2	0	2	0	2	2	2	0	2
Fractographic analysis	0	0	0	0	0	0	0	0	0	0	0	0	0	0	2	0	0	0	0	0	0	0	0	0	0	0	0	2	0
Manufacturer’s Instructions	0	0	0	0	0	0	0	0	0	0	0	0	0	0	0	0	0	0	0	0	0	0	0	0	0	0	0	0	0
Sample Size Calculation	0	2	0	0	0	0	0	2	0	2	0	2	0	2	2	2	0	0	2	2	2	0	2	0	2	2	2	2	2
International Standards	1	0	1	1	1	1	0	1	0	1	1	1	1	1	1	1	0	1	1	0	1	1	1	1	1	1	1	1	0
Proper statistical analysis	0	0	0	0	0	0	0	0	0	0	0	0	0	0	0	0	0	0	0	0	0	0	0	0	0	0	0	0	0
TOTAL	8	9	5	4	5	5	6	7	2	9	5	7	5	9	10	10	5	6	8	8	9	5	7	6	9	10	10	10	9
Risk of Bias	M	M	M	L	M	M	M	M	M	M	M	M	M	M	M	M	M	M	M	M	M	M	M	M	M	M	M	M	M
Journal SJR score by date of publication	Q2	Q2	Q2	Q2	Q2	Q2	Q1	Q2	Q1	Q1	Q1	Q1	Q1	Q1	Q1	Q2	Q1	Q2	Q1	Q1	Q1	Q1	Q3	Q1	Q1	Q2	Q1	Q2	Q2

**Table 2:** Resumé of extracted data from selected studies assessing adhesive strength.

Author, Year	Material	Sample Pairing	Type of Test	Surface Treatment	Coupling Agent	Adhesive System	Luting Cement
Abdou et al. (2021) [7]	Katana Avencia	Bovine incisors (n = 15)	μTBS	50 μm Al_2_O_3_ 37.5% PA	Kerr Silane primer SB-UA Clearfil Universal Bond Quick	Clearfil Universal Bond Quick SB-UA Optibond all-in-one	Panavia V5 Rely X Ultimate NX3 Nexus
Albelasy et al. (2021) [31]	IPS. e max CAD Vita Enamic Lava Ultimate	Human molars (n = 14)	Ultimate fracture test; thermocycling failure pattern	50 μm Al_2_O_3_ 8% HF 37% PA	Dentobond Silane	N/A	Rely X Unicem
Ali et al. (2012) [33]	Zirconia	Human molars (n = 12)	Load-to-fracture; thermocycling	50 μm Al_2_O_3_	N/A	ED primer	Panavia F 2.0 Rely X Unicem Clearfil SA
Augusti et al. (2020) [34]	Zirconia	Zirconia abutments (n = 10)	Pull-out test	50 μm Al_2_O_3_	N/A	N/A	Rely X Unicem 2
Bayazit et al. (2019) [8]	Lava Ultimate Vita Enamic	Blocks (n = 15)	μTBS	50 μm Al_2_O_3_ 9.5% HF	N/A	SB-UA	Rely X U200 Set PP
Ceci et al. (2016) [9]	Lava Ultimate	Bovine incisors (n = 10)	μSBS	50 μm Al_2_O_3_ 35% PA Clinpro powder (glycine)	SB-UA	SB-UA	Rely X Ultimate Rely X Unicem 2
Elsaka et al. (2014) [35]	Vita Enamic Lava Ultimate	Composite resin block (n = 3)	μTBS; aging	50 μm Al_2_O_3_ 9.5% HF	Silane	N/A	Bifix SE
Ender et al. (2016) [3]	IPS. Empress CAD ArtBlock Temp	Human molars (n = 12)	SBS	50 μm Al_2_O_3_	Monobond Plus	Heliobond	Rely X Unicem Variolink II
Higashi et al. (2016) [1]	Katana Avencia	Luting cement (n = 8)	μTBS; aging	50 μm Al_2_O_3_	Clearfil Ceramic Primer Plus	N/A	Panavia V5 Panavia SA
Kawaguchi et al. (2016) [10]	Katana Avencia	Luting cement (n = 8)	μTBS; aging	50 μm Al_2_O_3_ 40% PA K-Etchant gel	Clearfil Ceramic Primer Plus	N/A	Panavia V5 Panavia SA
Liebermann et al. (2013) [2]	ArtBlock temp	Luting cement (n = 20)	TBS; surface energy; surface roughness	50 μm Al_2_O_3_	N/A	Visiolink	Clearfil SA Rely X Unicem
Magne et al. (2015) [36]	Vita Mark II IPS. e max CAD Lava Ultimate	Human molars (n = 15)	Fatigue test	50 μm Al_2_O_3_ 27 μm Al_2_O_3_ 5% HF	Rely X Ceramic Primer	N/A	Rely X Unicem 2
Malysa et al. (2022) [37]	IPS Empress CAD IPS. e max CAD IPS. e max ZirCAD	Human Molars (n = 12)	SBS; load-to-fracture; thermocycling	9% HF 37% PA	N/A	N/A	Panavia V5 Maxcem Elite Rely X U200 Panavia SA
Nagasawa et al. (2021) [38]	Cerasmart Shofu Block HC HZR-CAD HR2 Estelite Vita Enamic Katana Avencia	Resin composite disk (n = 15)	SBS	70 μm Al_2_O_3_ 15-40% PA 9% HF	GC G-Multiprimer	N/A	G-Cem ONE
Nagasawa et al. (2022) [14]	GN I Ceramic Block Cerasmart	Resin composite disk (n = 15)	SBS	70 μm Al_2_O_3_	GC G-Multiprimer GC Ceramic Primer II	N/A	G-Cem ONE
Nakamura et al. (2016) [39]	Zirconia	Luting cement (n = 10) Crown (n = 6)	Load-to-failure test; micro-CT analysis	N/A	ED primer	N/A	RelyX Unicem 2 Panavia F2.0
Oda et al. (2021) [25]	Katana Avencia	Human molars (n = 5)	μTBS; irradiance measurements	50 μm Al_2_O_3_ 35% PA	Clearfil Ceramic Primer Plus	Clearfil SE Bond 2	Panavia SA Plus Panavia SA
Peumans et al. (2016) [40]	Celtra Duo IPS. e max CAD IPS Empress CAD Vita Enamic Vita Mark II Lava Ultimate	Block to block (n = 10)	μTBS	27 μm Al_2_O_3_ 30 μm Al_2_O_3_ <5% HF 600-grit Sic Paper Cojet-SiO_2_	Monobond Plus Heliobond	N/A	Clearfil Esthetic Panavia SA
Poggio et al. (2016) [6]	Lava Ultimate	Bovine incisors (n = 10)	SBS	35% PA	SB-UA	SB-UA	Rely X Ultimate Rely X Unicem 2
Preis et al. (2015) [4]	Celtra Duo IPS. e max CAD	Human molars (n = 8)	Thermal cycling and mechanical loading (chewing machine)	5% HF	Monobond S	Heliobond	Smart Cem 2 Variolink II
Sorrentino et al. (2016) [41]	Zirconia	Human molars (n = 10)	Load-to-fracture	50 μm Al_2_O_3_	N/A	N/A	G-Cem LinkAce
Takahashi et al. (2022) [42]	Estelite P Katana Avencia Shofu Black HC Super Hard	Luting cement (n = 10)	SBS	50 μm Al_2_O_3_	N/A	HC Primer	Panavia SA Block HC Cem
Ustun et al. (2021) [27]	Vita Suprinity Vita Enamic Cerasmart	Human molars (n = 7)	SBS; thermocycling	5% HF 37% PA	Ultradent Porcelain Silane	SB-UA	Rely X Ultimate Rely X U200
Zahoui et al. (2020) [43]	Zirconia	Ti-base CAD/CAM abutments (n = 10)	Pull-out test	30 μm Al_2_O_3_ 45 μm Al_2_O_3_	SB-UA	SB-UA	Rely X U200 Rely X Ultimate
Zhang et al. (2018) [51]	Zirconia	Luting cement (n = 20)	μSBS	50 μm Al_2_O_3_	SB-UA	SB-UA	Multilink Speed

Al_2_O_3_, aluminum oxide; HF, hydrofluoric acid; PA, phosphoric acid; N/A, not applied; SB-UA, Scotchbond Universal Adhesive; SiC, silica paper abrasive.

**Table 3:** Resumé of extracted data from selected studies assessing the marginal gap.

Author, Year	Material	Sample Pairing	Type of Test	Surface Treatment	Coupling Agent	Adhesive System	Luting Cement
Dauti et al. (2020) [5]	Vita Enamic	Model resin (n = 10)	Micro CT scan Marginal adaptation measurements	5% HF	Monobond Plus	AdheSE primer AdheSE Adhesive Optibond XTR OptiBond XTR Primer OptiBond XTR Bond	Rely X Unicem Variolink Esthetic NX3 Nexus
Ender et al. (2016) [3]	IPS. Empress CAD ArtBlock Temp	Human molars (n = 12)	Marginal adaptation, chewing fatigue test	50 μm Al_2_O_3_	Monobond Plus	Heliobond	Rely X Unicem Variolink II
Han et al. (2020) [28]	Lava Ultimate	Human molars (n = 6)	Thermocycling; interfacial adaptation	50 μm Al_2_O_3_ Polyacrylic acid	N/A	Universal dentine adhesive Clearfil Universal bond quick Ceramic Primer Plus	Panavia V5 Rely X U200 G-Cem LinkAce SmartCem2 Multilink speed
Kirsten et al. (2018) [26]	Vita Mark II	Human molars (n = 8)	Evaluation of crown integrity and cement gap thickness	35 μm Al_2_O_3_ 37% PA 5% HF	N/A	Syntac	iCEM Rely X Unicem 2 Variolink Esthetic
Melo Freire et al. (2017) [22]	IPS. e max CAD IPS. e max Press	Bovine teeth (n = 64)	Marginal adaptation SEM	10% HF 35% PA	Rely X Ceramic Primer	Adper Single Bond Plus	Rely X ARC Rely X U200
Preis et al. (2015) [4]	Celtra Duo IPS. e max CAD	Human molars (n = 8)	Thermocycling; marginal quality (i) intact margin (ii) marginal gap	5% HF	Monobond S	Heliobond	Smart Cem 2 Variolink II

Al_2_O_3_, aluminum oxide; HF, hydrofluoric acid; PA, phosphoric acid; N/A, not applied.

**Table 4:** Synopsis of the CAD-CAM ceramic blocks by type of material based on the manufacturer’s official datasheet.

Material	Type of Material	Physical Properties	Manufacturer
ArtBlock Temp	Bis-acrylic composite blocks for temporary crowns and bridges. Highly cross-linked interpenetrated PMMA, the OMP-N (organic modified polymer network), without inorganic fillers	Flexural strength: > 90 MPa Module of elasticity: 2.680 MPa Organic curing agent OMP-N Does not contain inorganic fillers	Merz Dental GmbH, Germany
Celtra Duo	Zirconia-reinforced lithium silicate ceramic	Median load fracture: 725 N Fracture toughness: 2.6 MPa·m^1/2^	Dentsply Sirona, Germany
Cerasmart	Hybrid ceramic composite	Flexural strength: 238 MPa Breaking energy: 2.2 N/cm Preserved marginal integrity	GC Corporation, Japan
Estelite	Submicron-filled composite	Flexural strength: 259 MPa Elastic modulus: 13.8 GPa	Tokuyama Dental Corporation, Japan
GN I Ceramic Block	Hybrid ceramic composite material with inorganic fillers (silica, zirconia, and alumina)	Flexural strength: > 500 MPa Low thermal conductivity Color stability	GC Corporation, Japan
HZR-CAD HR2	Hybrid ceramic with ceramic cluster filler (1–20 µm)	Flexural strength: > 250 MPa Sustained fluoride release High abrasion resistance	Yamakin, Japan
IPS Empress CAD	Lithium disilicate glass-ceramic	Biaxial flexural strength: 185 MPa	Ivoclar Vivadent, Liechtenstein
IPS. e max CAD		Biaxial flexural strength: 530 MPa Fracture toughness: 2.11 MPa·m^1/2^ Rapid crystallization: 11 min	
IPS. e max Press		Flexural strength: 470 MPa Fracture toughness: 2.5-3 MPa·m^1/2^	
IPS. e max ZirCAD	Zirconium oxide	Flexural strength: 850–1200 MPa	
Katana Avencia	Hybrid ceramic (nanosized fillers densely compressed into block and infused with resin monomer)	Flexural strength: > 220 MPa Compressive strength: > 600 MPa Excellent wear resistance	Kuraray Noritake, Japan
Lava Ultimate	Highly cross-linked polymeric matrix embedded with 80% of nanoceramic components	Elastic modulus similar to dentin High resistance to fracture	3M ESPE, USA
Shofu Block HC	Pre-sintered, highly filled hybrid ceramic block made of zirconia-reinforced lithium silicate	Stress-absorbing hybrid-ceramic material Flexural strength: > 190 MPa Excellent handling and milling properties	SHOFU Dental GmbH, Japan
Vita Enamic	Hybrid ceramic with a dual ceramic-polymer network structure	Flexural strength: ± 160 MPa Module of elasticity: 3 MPa Fracture toughness: 1.5 MPa·m^1/2^	Vita Zahnfabrik, Germany
Vita Mark II	Fine-structure (4 µm) feldspar ceramic	Flexural strength: 150-160 MPa Elastic modulus: 30.0 GPa Static fracture load: 2.766 N	
Vita Suprinity	High-strength zirconia-reinforced lithium silicate ceramic material	Flexural strength: ± 420 MPa Module of elasticity: 7 MPa Fracture toughness: ± 2.0 MPa·m^1/2^	
Zenostar	Zirconium oxide	Flexural strength > 900 MPa Good abrasive characteristics Gingiva-friendly	Wieland, Germany

**Table 5:** CAD-CAM blocks identified in articles for quantitative analysis.

Material	Frequency	Percent	Cumulative
Artblock Temp	12	4.48	4.58
Cerasmart	28	10.69	15.27
Estelite block	13	4.96	20.23
HZR-CAD-HR2	5	1.91	22.14
IPS Empress CAD	12	4.58	26.72
IPS e. max Zircad	12	4.58	31.30
IPS e. max CAD	12	4.58	35.88
Katana Avencia	97	37.02	72.90
Lava Ultimate	25	9.54	82.44
Shofu Block Hc	13	4.96	87.40
Vita Enamic	27	10.31	97.71
Vita Suprinity	6	2.29	100.00
Total	262	100.00	

## Data Availability

Not applicable.

## Supplementary Materials

The following supporting information can be downloaded at: https://www.mdpi.com/article/10.3390/ma16082996/s1, Table S1: Effect size calculation.
