# Supplementary material for: Effectiveness of Self-Adhesive Resin Luting Cement in CAD-CAM Blocks—A Systematic Review and Meta-Analysis
Source: Materials (Basel). 2023 Apr 10;16(8):2996. doi: 10.3390/ma16082996 (PMC10140979; doi:10.3390/ma16082996)
Supplement: Supplementary file 1 [file materials-16-02996-s001.zip › materials-2277936-supplementary.pdf]

(From Hedges and Olkin p80)

Author, year	material	surface treatment	aging	coupling agent adhesive system	luting cement	Test (MPa)	mean	n	SD	I_mean	I_n	I_SD	pooled_SD	p-value_diff_SDs	mean_diff	p-value_mean_diff	lower_CI	upper_CI	eS	Bias_corrected_Hedges	SD_eS	lower_eS-CI	upper_eS-CI	eS_lowmean			n-2	bias correction factor	approx
Abdou et al (2021) [7]	Katana Avencia	PA 37%	1 week water	Kerr Silane Primer	Nexus 3	μTBS	26.10	5	4.60	26.10	5	4.60	4.60	0.50	0.00	1.00	-6.71	6.71	0.00	0.00	0.63	-1.24	1.24	0.00			2	0.5642	0.571428571
Abdou et al (2021) [7]	Katana Avencia	PA 37%	1 week water	Kerr Silane Primer	Nexus 3	μTBS	27.70	5	3.80	26.10	5	4.60	4.22	0.36	1.60	0.57	-4.55	7.75	0.38	0.34	0.64	-0.91	1.59	0.35			3	0.7236	0.727272727
Abdou et al (2021) [7]	Katana Avencia	PA 37%	1 hour water	Kerr Silane Primer	Nexus 3	μTBS	27.20	5	4.10	26.10	5	4.60	4.36	0.41	1.10	0.70	-5.25	7.45	0.25	0.23	0.63	-1.02	1.47	0.24			4	0.7979	0.8
Abdou et al (2021) [7]	Katana Avencia	PA 37%	1 hour water	Kerr Silane Primer	Nexus 3	μTBS	29.30	5	2.20	26.10	5	4.60	3.61	0.09	3.20	0.20	-2.06	8.46	0.89	0.80	0.66	-0.49	2.09	0.70			5	0.8408	0.842105263
Abdou et al (2021) [7]	Katana Avencia	Al2O3 50 μm	1 hour water	Clearfil Univ. Bond Quick	Panavia V5	μTBS	48.30	5	4.40	29.30	5	2.40	3.54	0.13	19.00	0.00	13.83	24.17	5.36	4.84	1.25	2.38	7.30	7.92			6	0.8686	0.869565217
Abdou et al (2021) [7]	Katana Avencia	Al2O3 50 μm	1 hour water	Clearfil Univ. Bond Quick	Panavia V5	μTBS	39.40	5	2.80	29.30	5	2.40	2.61	0.39	10.10	0.00	6.30	13.90	3.87	3.50	1.01	1.53	5.47	4.21	% confidence interval:	95	7	0.8882	0.888888889
Abdou et al (2021) [7]	Katana Avencia	Al2O3 50 μm	1 week water	Clearfil Univ. Bond Quick	Panavia V5	μTBS	37.20	5	1.70	29.30	5	2.40	2.08	0.26	7.90	0.00	4.87	10.93	3.80	3.43	0.99	1.48	5.38	3.29	p:	0.05	8	0.9027	0.903225806
Abdou et al (2021) [7]	Katana Avencia	Al2O3 50 μm	1 hour water	SB-UA	Rely X Ultimate	μTBS	36.40	5	2.40	27.70	5	3.80	3.18	0.20	8.70	0.00	4.06	13.34	2.74	2.47	0.84	0.83	4.12	2.29	Z-value:	1.95996	9	0.9139	0.914285714
Abdou et al (2021) [7]	Katana Avencia	Al2O3 50 μm	1 week water	SB-UA	Rely X Ultimate	μTBS	31.30	5	2.50	27.70	5	3.80	3.22	0.22	3.60	0.11	-1.09	8.29	1.12	1.01	0.67	-0.31	2.33	0.95			10	0.9228	0.923076923
Abdou et al (2021) [7]	Katana Avencia	Al2O3 50 μm	1 hour water	SB-UA	Rely X Ultimate	μTBS	44.00	5	3.40	27.70	5	3.80	3.61	0.42	16.30	0.00	11.04	21.56	4.52	4.08	1.11	1.90	6.26	4.29			11	0.93	0.930232558
Bayazit et al (2019) [8]	Lava Ultimate	HA 9.5% % + UA	24 hours 37ºC water	SB-UA	Rely X U200	μTBS	38.70	15	6.40	17.60	15	4.60	5.57	0.11	21.10	0.00	16.93	25.27	3.79	3.68	0.60	2.51	4.86	4.59			12	0.94	0.936170213
Bayazit et al (2019) [8]	Lava Ultimate	Al2O3 50μm + UA	24 hours 37ºC water	SB-UA	Rely X U200	μTBS	31.70	15	4.50	17.60	15	4.60	4.55	0.47	14.10	0.00	10.70	17.50	3.10	3.01	0.53	1.97	4.06	3.07			13	0.941	0.941176471
Bayazit et al (2019) [8]	Lava Ultimate	NO	24 hours 37ºC water	NO	Rely X U200	μTBS	19.10	15	4.90	17.60	15	4.60	4.75	0.41	1.50	0.39	-2.05	5.05	0.32	0.31	0.37	-0.41	1.03	0.33			14	0.9453	0.945454545
Bayazit et al (2019) [8]	Vita Enamic	Al2O3 50μm + UA	24 hours 37ºC water	SB-UA	Rely X U200	μTBS	30.80	15	5.40	17.60	15	4.60	5.02	0.28	13.20	0.00	9.45	16.95	2.63	2.56	0.49	1.59	3.53	2.87			15	0.949	0.949152542
Bayazit et al (2019) [8]	Vita Enamic	HA 9.5% % + UA	24 hours 37ºC water	SB-UA	Rely X U200	μTBS	23.70	15	4.30	17.60	15	4.60	4.45	0.40	6.10	0.00	2.77	9.43	1.37	1.33	0.40	0.54	2.12	1.33			16	0.9523	0.952380952
Bayazit et al (2019) [8]	Lava Ultimate	Al2O3 50 μm + UA	24 hours 37ºC water	SB-UA	Set PP	μTBS	25.30	15	5.00	18.90	15	5.90	5.47	0.27	6.40	0.00	2.31	10.49	1.17	1.14	0.39	0.37	1.91	1.08			17	0.9551	0.955223881
Bayazit et al (2019) [8]	Lava Ultimate	NO	24 hours 37ºC water	SB-UA	Set PP	μTBS	22.10	15	4.60	18.90	15	5.90	5.29	0.18	3.20	0.11	-0.76	7.16	0.60	0.59	0.37	-0.14	1.32	0.54			18	0.9577	0.957746479
Bayazit et al (2019) [8]	Lava Ultimate	HA 9.5% % + UA	24 hours 37ºC water	SB-UA	Set PP	μTBS	28.30	15	4.20	18.90	15	5.90	5.12	0.11	9.40	0.00	5.57	13.23	1.84	1.79	0.43	0.94	2.63	1.59			19	0.9599	0.96
Bayazit et al (2019) [8]	Vita Enamic	Al2O3 50 μm + UA	24 hours 37ºC water	SB-UA	Set PP	μTBS	18.90	15	5.90	18.90	15	5.90	5.90	0.50	0.00	1.00	-4.41	4.41	0.00	0.00	0.37	-0.72	0.72	0.00			20	0.9619	0.962025316
Bayazit et al (2019) [8]	Vita Enamic	HA 9.5% % + UA	24 hours 37ºC water	SB-UA	Set PP	μTBS	20.90	15	3.80	18.90	15	5.90	4.96	0.06	2.00	0.28	-1.71	5.71	0.40	0.39	0.37	-0.33	1.11	0.34			21	0.9638	0.963855422
Bayazit et al (2019) [8]	Vita Enamic	NO	24 hours 37ºC water	NO	Set PP	μTBS	19.80	15	2.80	18.90	15	5.90	4.62	0.00	0.90	0.60	-2.55	4.35	0.19	0.19	0.37	-0.53	0.91	0.15			22	0.9655	0.965517241
Ceci et al (2016) [9]	Lava Ultimate	Glycine + Al2O3 50 μm	24 hours water	SB-UA	Rely X Ultimate	μSBS	17.88	5	1.44	9.98	5	1.94	1.71	0.29	7.90	0.00	5.41	10.39	4.62	4.17	1.13	1.96	6.38	4.07			23	0.967	0.967032967
Ceci et al (2016) [9]	Lava Ultimate	Al2O3 50 μm + 35% PA	24 hours water	SB-UA	Rely X Ultimate	μSBS	11.98	5	2.91	9.98	5	1.94	2.47	0.23	2.00	0.24	-1.61	5.61	0.81	0.73	0.65	-0.55	2.01	1.03			24	0.9684	0.968421053
Ceci et al (2016) [9]	Lava Ultimate	Al2O3 50 μm	24 hours water	SB-UA	Rely X Ultimate	μSBS	16.58	5	3.87	9.98	5	1.94	3.06	0.10	6.60	0.01	2.14	11.06	2.16	1.95	0.77	0.44	3.45	3.40			25	0.9699	0.96969697
Elsaka et al (2014) [35]	Lava Ultimate	HF 9%	24 hours water	NO	Bifix SE	μTBS	18.35	3	2.84	11.99	3	2.52	2.68	0.44	6.36	0.04	0.27	12.45	2.37	1.89	0.98	-0.03	3.81	2.52					
Elsaka et al (2014) [35]	Lava Ultimate	Al2O3 50 μm	30 days water	NO	Bifix SE	μTBS	13.49	3	3.40	11.99	3	2.52	2.99	0.35	1.50	0.57	-5.28	8.28	0.50	0.40	0.82	-1.22	2.02	0.60			26	0.9708	0.970873786
Elsaka et al (2014) [35]	Lava Ultimate	Al2O3 50 μm	24 hours water	NO	Bifix SE	μTBS	16.93	3	3.66	11.99	3	2.52	3.14	0.32	4.94	0.13	-2.18	12.06	1.57	1.25	0.89	-0.50	3.01	1.96			27	0.9719	0.971962617
Elsaka et al (2014) [35]	Lava Ultimate	HF 9% + SILANE	24 hours water	Ultradent Silane	Bifix SE	μTBS	19.21	3	3.87	11.99	3	2.52	3.27	0.30	7.22	0.05	-0.18	14.62	2.21	1.76	0.96	-0.12	3.65	2.87			28	0.9729	0.972972973
Elsaka et al (2014) [35]	Lava Ultimate	HF 9%	30 days water	NO	Bifix SE	μTBS	13.45	3	3.34	11.99	3	2.52	2.96	0.36	1.46	0.58	-5.25	8.17	0.49	0.39	0.82	-1.22	2.01	0.58			29	0.9739	0.973913043
Elsaka et al (2014) [35]	Lava Ultimate	HF 9% + SILANE	30 days water	Ultradent Silane	Bifix SE	μTBS	14.35	3	2.56	11.99	3	2.52	2.54	0.49	2.36	0.32	-3.40	8.12	0.93	0.74	0.84	-0.91	2.40	0.94			30	0.9748	0.974789916
Elsaka et al (2014) [35]	Lava Ultimate	Al2O3 50 μm + Silane	30 days water	NO	Bifix SE	μTBS	13.88	3	3.47	11.99	3	2.52	3.03	0.35	1.89	0.49	-4.98	8.76	0.62	0.50	0.83	-1.13	2.12	0.75			31	0.9756	0.975609756
Elsaka et al (2014) [35]	Lava Ultimate	Al2O3 50 μm + Silane	24 hours water	Ultradent Silane	Bifix SE	μTBS	18.82	3	3.69	11.99	3	2.52	3.16	0.32	6.83	0.06	-0.33	13.99	2.16	1.72	0.96	-0.15	3.60	2.71			32	0.9764	0.976377953
Elsaka et al (2014) [35]	Lava Ultimate	No treatment	30 days water	NO	Bifix SE	μTBS	8.28	3	1.99	11.99	3	2.52	2.27	0.38	3.71	0.12	-1.44	8.86	1.63	1.30	0.90	-0.46	3.07	1.47			33	0.9771	0.977099237
Elsaka et al (2014) [35]	Vita Enamic	No treatment	24 hours water	NO	Bifix SE	μTBS	18.67	3	3.10	11.99	3	2.52	2.82	0.40	6.68	0.04	0.28	13.08	2.36	1.89	0.98	-0.04	3.81	2.65			34	0.9778	0.977777778
Elsaka et al (2014) [35]	Vita Enamic	HF 9% + SILANE	24 hours water	Ultradent Silane	Bifix SE	μTBS	27.47	3	4.28	11.99	3	2.52	3.51	0.26	15.48	0.01	7.52	23.44	4.41	3.52	1.30	0.96	6.07	6.14			36	0.979	0.979020979

Elsaka et al (2014) [35]	Vita Enamic	HF 9%	24 hours water	NO	Bifix SE	µTBS	23.86	3	3.19	11.99	3	2.52	2.87	0.38	11.87	0.01	5.35	18.39	4.13	3.29	1.25	0.84	5.75	4.71	37	0.9796	0.979591837
Elsaka et al (2014) [35]	Vita Enamic	Al2O3 50 µm + Silane	30 days water	Ultradent Silane	Bifix SE	µTBS	19.48	3	3.18	11.99	3	2.52	2.87	0.39	7.49	0.03	0.99	13.99	2.61	2.08	1.01	0.10	4.07	2.97	38	0.9801	0.98013245
Elsaka et al (2014) [35]	Vita Enamic	No treatment	30 days water	NO	Bifix SE	µTBS	12.67	3	2.13	11.99	3	2.52	2.33	0.42	0.68	0.74	-4.61	5.97	0.29	0.23	0.82	-1.37	1.84	0.27	39	0.9806	0.980645161
Elsaka et al (2014) [35]	Vita Enamic	Al2O3 50 µm + Silane	24 hours water	Ultradent Silane	Bifix SE	µTBS	24.95	3	3.79	11.99	3	2.52	3.22	0.31	12.96	0.01	5.66	20.26	4.03	3.21	1.24	0.79	5.64	5.14	40	0.9811	0.981132075
Elsaka et al (2014) [35]	Vita Enamic	HF 9%	30 days water	NO	Bifix SE	µTBS	18.86	3	3.31	11.99	3	2.52	2.94	0.37	6.87	0.05	0.20	13.54	2.34	1.86	0.98	-0.05	3.78	2.73	41	0.9816	0.981595092
Elsaka et al (2014) [35]	Vita Enamic	Al2O3 50 µm	24 hours water	NO	Bifix SE	µTBS	21.87	3	3.75	11.99	3	2.52	3.19	0.31	9.88	0.02	2.64	17.12	3.09	2.47	1.08	0.34	4.59	3.92	42	0.982	0.982035928
Elsaka et al (2014) [35]	Vita Enamic	Al2O3 50 µm	30 days water	NO	Bifix SE	µTBS	16.71	3	3.42	11.99	3	2.52	3.00	0.35	4.72	0.13	-2.09	11.53	1.57	1.25	0.89	-0.50	3.00	1.87	43	0.9824	0.98245614
Elsaka et al (2014) [35]	Vita Enamic	HF 9% + SILANE	30 days water	Ultradent Silane	Bifix SE	µTBS	22.21	3	3.04	11.99	3	2.52	2.79	0.41	10.22	0.01	3.89	16.55	3.66	2.92	1.17	0.62	5.22	4.06	44	0.9828	0.982857143
Higashi et al (2016) [1]	Katana Avencia	Al2O3 50 µm	No water	NO	Panavia SA	µTBS	66.02	3	10.67	25.03	3	12.34	11.54	0.43	40.99	0.01	14.84	67.14	3.55	2.84	1.16	0.57	5.10	3.32	45	0.9832	0.983240223
Higashi et al (2016) [1]	Katana Avencia	No treatment	3 months water	NO	Panavia SA	µTBS	26.05	3	7.66	25.03	3	12.34	10.27	0.28	1.02	0.91	-22.26	24.30	0.10	0.08	0.82	-1.52	1.68	0.08	46	0.9836	0.983606557
Higashi et al (2016) [1]	Katana Avencia	Al2O3 50 µm	3 months water	NO	Panavia SA	µTBS	65.75	3	15.37	25.03	3	12.34	13.94	0.39	40.72	0.02	9.12	72.32	2.92	2.33	1.06	0.26	4.40	3.30	47	0.9839	0.983957219
Higashi et al (2016) [1]	Katana Avencia	Al2O3 50 µm + Silane	No water	Clearfil Cer. Primer Plus	Panavia SA	µTBS	59.31	3	13.44	25.03	3	12.34	12.90	0.46	34.28	0.03	5.03	63.53	2.66	2.12	1.02	0.12	4.12	2.78	48	0.9843	0.984293194
Higashi et al (2016) [1]	Katana Avencia	No treatment	No water	NO	Panavia SA	µTBS	40.42	3	11.36	25.03	3	12.34	11.86	0.46	15.39	0.19	-11.50	42.28	1.30	1.04	0.87	-0.67	2.74	1.25	49	0.9846	0.984615385
Higashi et al (2016) [1]	Katana Avencia	Al2O3 50 µm + Silane	3 months water	Clearfil Cer. Primer Plus	Panavia SA	µTBS	60.04	3	15.93	25.03	3	12.34	14.25	0.38	35.01	0.04	2.71	67.31	2.46	1.96	0.99	0.01	3.91	2.84	50	0.9849	0.984924623
Higashi et al (2016) [1]	Katana Avencia	SILANE	No water	Clearfil Cer. Primer Plus	Panavia SA	µTBS	70.95	3	11.90	25.03	3	12.34	12.12	0.48	45.92	0.01	18.44	73.40	3.79	3.02	1.19	0.68	5.36	3.72	52	0.9855	0.985507246
Higashi et al (2016) [1]	Katana Avencia	SILANE	3 months water	Clearfil Cer. Primer Plus	Panavia SA	µTBS	55.71	3	9.91	25.03	3	12.34	11.19	0.39	30.68	0.03	5.31	56.05	2.74	2.19	1.03	0.16	4.21	2.49	54	0.986	0.986046512
Higashi et al (2016) [1]	Katana Avencia	No treatment	1 month water	NO	Panavia SA	µTBS	33.02	3	16.45	25.03	3	12.34	14.54	0.36	7.99	0.54	-24.97	40.95	0.55	0.44	0.83	-1.18	2.06	0.65	56	0.9865	0.986547085
Higashi et al (2016) [1]	Katana Avencia	SILANE	6 months water	Clearfil Cer. Primer Plus	Panavia SA	µTBS	54.24	3	11.44	25.03	3	12.34	11.90	0.46	29.21	0.04	2.24	56.18	2.45	1.96	0.99	0.01	3.91	2.37	58	0.987	0.987012987
Higashi et al (2016) [1]	Katana Avencia	Al2O3 50 µm + Silane	1 month water	Clearfil Cer. Primer Plus	Panavia SA	µTBS	55.59	3	14.23	25.03	3	12.34	13.32	0.43	30.56	0.05	0.37	60.75	2.29	1.83	0.97	-0.08	3.74	2.48	60	0.9874	0.987447699
Higashi et al (2016) [1]	Katana Avencia	Al2O3 50 µm	6 months water	NO	Panavia SA	µTBS	59.44	3	20.40	25.03	3	12.34	16.86	0.27	34.41	0.07	-3.81	72.63	2.04	1.63	0.94	-0.22	3.48	2.79	62	0.9879	0.987854251
Higashi et al (2016) [1]	Katana Avencia	SILANE	1 month water	Clearfil Cer. Primer Plus	Panavia SA	µTBS	64.29	3	15.87	25.03	3	12.34	14.22	0.38	39.26	0.03	7.04	71.48	2.76	2.20	1.04	0.18	4.23	3.18	64	0.9882	0.988235294
Higashi et al (2016) [1]	Katana Avencia	Al2O3 50 µm	1 month water	NO	Panavia SA	µTBS	68.46	3	19.06	25.03	3	12.34	16.06	0.30	43.43	0.03	7.03	79.83	2.70	2.16	1.03	0.15	4.17	3.52	66	0.9886	0.988593156
Higashi et al (2016) [1]	Katana Avencia	Al2O3 50 µm + Silane	6 months water	Clearfil Cer. Primer Plus	Panavia SA	µTBS	56.44	3	17.88	25.03	3	12.34	15.36	0.32	31.41	0.07	-3.41	66.23	2.04	1.63	0.94	-0.22	3.48	2.55	68	0.9889	0.988929889
Higashi et al (2016) [1]	Katana Avencia	Al2O3 50 µm	6 months water	NO	Panavia v5	µTBS	82.41	3	27.64	24.02	3	9.59	20.69	0.11	58.39	0.03	11.49	105.29	2.82	2.25	1.04	0.21	4.30	6.09	70	0.9892	0.989247312
Higashi et al (2016) [1]	Katana Avencia	No treatment	3 months water	NO	Panavia v5	µTBS	23.15	3	12.39	24.02	3	9.59	11.08	0.37	0.87	0.93	-24.25	25.99	0.08	0.06	0.82	-1.54	1.66	0.09	72	0.9895	0.989547038
Higashi et al (2016) [1]	Katana Avencia	Al2O3 50 µm	1 month water	NO	Panavia v5	µTBS	63.51	3	13.37	24.02	3	9.59	11.63	0.34	39.49	0.01	13.11	65.87	3.39	2.71	1.13	0.49	4.92	4.12	74	0.9898	0.989830508
Higashi et al (2016) [1]	Katana Avencia	Al2O3 50 µm + Silane	1 month water	Clearfil Cer. Primer Plus	Panavia v5	µTBS	84.24	3	11.43	24.02	3	9.59	10.55	0.41	60.22	0.00	36.30	84.14	5.71	4.55	1.55	1.52	7.59	6.28	76	0.9901	0.99009901
Higashi et al (2016) [1]	Katana Avencia	Al2O3 50 µm	No water	NO	Panavia v5	µTBS	78.40	3	12.65	24.02	3	9.59	11.22	0.36	54.38	0.00	28.93	79.83	4.84	3.87	1.38	1.16	6.58	5.67			
Higashi et al (2016) [1]	Katana Avencia	Al2O3 50 µm + Silane	3 months water	Clearfil Cer. Primer Plus	Panavia v5	µTBS	80.73	3	18.22	24.02	3	9.59	14.56	0.22	56.71	0.01	23.71	89.71	3.90	3.11	1.21	0.73	5.49	5.91			
Higashi et al (2016) [1]	Katana Avencia	SILANE	6 months water	Clearfil Cer. Primer Plus	Panavia v5	µTBS	59.60	3	13.10	24.02	3	9.59	11.48	0.35	35.58	0.02	9.56	61.60	3.10	2.47	1.08	0.35	4.60	3.71			
Higashi et al (2016) [1]	Katana Avencia	Al2O3 50 µm + Silane	No water	Clearfil Cer. Primer Plus	Panavia v5	µTBS	86.98	3	6.40	24.02	3	9.59	8.15	0.31	62.96	0.00	44.48	81.44	7.72	6.16	1.96	2.33	10.00	6.57			
Higashi et al (2016) [1]	Katana Avencia	SILANE	3 months water	Clearfil Cer. Primer Plus	Panavia v5	µTBS	66.73	3	13.65	24.02	3	9.59	11.80	0.33	42.71	0.01	15.97	69.45	3.62	2.89	1.17	0.60	5.18	4.45			
Higashi et al (2016) [1]	Katana Avencia	Al2O3 50 µm + Silane	6 months water	Clearfil Cer. Primer Plus	Panavia v5	µTBS	79.42	3	11.42	24.02	3	9.59	10.54	0.41	55.40	0.00	31.50	79.30	5.25	4.19	1.46	1.33	7.05	5.78			
Higashi et al (2016) [1]	Katana Avencia	SILANE	No water	Clearfil Cer. Primer Plus	Panavia v5	µTBS	82.51	3	12.36	24.02	3	9.59	11.06	0.38	58.49	0.00	33.41	83.57	5.29	4.22	1.47	1.35	7.09	6.10			
Higashi et al (2016) [1]	Katana Avencia	SILANE	1 month water	Clearfil Cer. Primer Plus	Panavia v5	µTBS	78.59	3	13.57	24.02	3	9.59	11.75	0.33	54.57	0.00	27.93	81.21	4.64	3.71	1.35	1.07	6.34	5.69			
Higashi et al (2016) [1]	Katana Avencia	No treatment	No water	NO	Panavia v5	µTBS	37.19	3	13.33	24.02	3	9.59	11.61	0.34	13.17	0.24	-13.15	39.49	1.13	0.90	0.86	-0.78	2.59	1.37			
Higashi et al (2016) [1]	Katana Avencia	No treatment	6 months water	NO	Panavia v5	µTBS	25.10	3	13.57	24.02	3	9.59	11.75	0.33	1.08	0.92	-25.56	27.72	0.09	0.07	0.82	-1.53	1.67	0.11			
Higashi et al (2016) [1]	Katana Avencia	Al2O3 50 µm	3 months water	NO	Panavia v5	µTBS	76.18	3	15.53	24.02	3	9.59	12.91	0.28	52.16	0.01	22.90	81.42	4.04	3.22	1.24	0.80	5.65	5.44			
Kawaguchi et al (2016) [10]	Katana Avencia	PA 40%	6 months water	Clearfil Cer. Primer Plus	Panavia SA	µTBS	46.10	3	14.30	46.70	3	10.90	12.71	0.37	0.60	0.96	-28.22	29.42	0.05	0.04	0.82	-1.56	1.64	0.06			

Kawaguchi et al (2016) [10]	Katana Avencia	Ultrass. cleaning + PA 40%	No water	Clearfil Cer. Primer Plus	Panavia SA	µTBS	63.90	3	10.90	46.70	3	10.90	10.90	0.50	17.20	0.13	-7.51	41.91	1.58	1.26	0.89	-0.49	3.01	1.58
Kawaguchi et al (2016) [10]	Katana Avencia	Ultrass. cleaning	1 month water	Clearfil Cer. Primer Plus	Panavia SA	µTBS	55.30	3	9.40	46.70	3	10.90	10.18	0.43	8.60	0.36	-14.47	31.67	0.84	0.67	0.84	-0.97	2.32	0.79
Kawaguchi et al (2016) [10]	Katana Avencia	PA 40%	1 month water	Clearfil Cer. Primer Plus	Panavia SA	µTBS	62.40	3	8.20	46.70	3	10.90	9.64	0.36	15.70	0.12	-6.16	37.56	1.63	1.30	0.90	-0.46	3.06	1.44
Kawaguchi et al (2016) [10]	Katana Avencia	PA 40%	No water	Clearfil Cer. Primer Plus	Panavia SA	µTBS	62.60	3	9.90	46.70	3	10.90	10.41	0.45	15.90	0.13	-7.70	39.50	1.53	1.22	0.89	-0.52	2.96	1.46
Kawaguchi et al (2016) [10]	Katana Avencia	No cleaning treatment	3 months water	Clearfil Cer. Primer Plus	Panavia SA	µTBS	54.00	3	10.90	46.70	3	10.90	10.90	0.50	7.30	0.46	-17.41	32.01	0.67	0.53	0.83	-1.09	2.16	0.67
Kawaguchi et al (2016) [10]	Katana Avencia	No cleaning treatment	6 months water	Clearfil Cer. Primer Plus	Panavia SA	µTBS	48.80	3	10.00	46.70	3	10.90	10.46	0.46	2.10	0.82	-21.61	25.81	0.20	0.16	0.82	-1.44	1.76	0.19
Kawaguchi et al (2016) [10]	Katana Avencia	Ultrass. cleaning	3 months water	Clearfil Cer. Primer Plus	Panavia SA	µTBS	55.80	3	11.30	46.70	3	10.90	11.10	0.48	9.10	0.37	-16.07	34.27	0.82	0.65	0.84	-0.99	2.30	0.83
Kawaguchi et al (2016) [10]	Katana Avencia	PA 40%	3 months water	Clearfil Cer. Primer Plus	Panavia SA	µTBS	49.50	3	16.50	46.70	3	10.90	13.98	0.30	2.80	0.82	-28.90	34.50	0.20	0.16	0.82	-1.44	1.76	0.26
Kawaguchi et al (2016) [10]	Katana Avencia	Ultrass. cleaning + PA 40%	1 month water	Clearfil Cer. Primer Plus	Panavia SA	µTBS	62.90	3	12.50	46.70	3	10.90	11.73	0.43	16.20	0.17	-10.39	42.79	1.38	1.10	0.88	-0.62	2.82	1.49
Kawaguchi et al (2016) [10]	Katana Avencia	No cleaning treatment	1 month water	Clearfil Cer. Primer Plus	Panavia SA	µTBS	60.90	3	11.00	46.70	3	10.90	10.95	0.50	14.20	0.19	-10.62	39.02	1.30	1.03	0.87	-0.67	2.74	1.30
Kawaguchi et al (2016) [10]	Katana Avencia	Ultrass. cleaning	No water	Clearfil Cer. Primer Plus	Panavia SA	µTBS	62.60	3	9.90	46.70	3	10.90	10.41	0.45	15.90	0.13	-7.70	39.50	1.53	1.22	0.89	-0.52	2.96	1.46
Kawaguchi et al (2016) [10]	Katana Avencia	Ultrass. cleaning	6 months water	Clearfil Cer. Primer Plus	Panavia SA	µTBS	48.80	3	10.50	46.70	3	10.90	10.70	0.48	2.10	0.82	-22.16	26.36	0.20	0.16	0.82	-1.45	1.76	0.19
Kawaguchi et al (2016) [10]	Katana Avencia	Ultrass. cleaning + PA 40%	6 months water	Clearfil Cer. Primer Plus	Panavia SA	µTBS	46.70	3	13.10	46.70	3	10.90	12.05	0.41	0.00	1.00	-27.32	27.32	0.00	0.00	0.82	-1.60	1.60	0.00
Kawaguchi et al (2016) [10]	Katana Avencia	No cleaning treatment	No water	Clearfil Cer. Primer Plus	Panavia SA	µTBS	62.70	3	10.40	46.70	3	10.90	10.65	0.48	16.00	0.14	-8.15	40.15	1.50	1.20	0.89	-0.54	2.94	1.47
Kawaguchi et al (2016) [10]	Katana Avencia	PA 40%	No water	Clearfil Cer. Primer Plus	Panavia v5	µTBS	81.10	3	7.60	70.00	3	13.90	11.20	0.23	11.10	0.29	-14.29	36.49	0.99	0.79	0.85	-0.87	2.45	0.80
Kawaguchi et al (2016) [10]	Katana Avencia	No cleaning treatment	1 month water	Clearfil Cer. Primer Plus	Panavia v5	µTBS	98.10	3	11.50	70.00	3	13.90	12.76	0.41	28.10	0.05	-0.82	57.02	2.20	1.76	0.96	-0.13	3.64	2.02
Kawaguchi et al (2016) [10]	Katana Avencia	Ultrass. cleaning + PA 40%	6 months water	Clearfil Cer. Primer Plus	Panavia v5	µTBS	65.90	3	14.60	70.00	3	13.90	14.25	0.48	4.10	0.74	-28.21	36.41	0.29	0.23	0.82	-1.38	1.84	0.29
Kawaguchi et al (2016) [10]	Katana Avencia	Ultrass. cleaning + PA 40%	3 months water	Clearfil Cer. Primer Plus	Panavia v5	µTBS	70.10	3	10.00	70.00	3	13.90	12.11	0.34	0.10	0.99	-27.35	27.55	0.01	0.01	0.82	-1.59	1.61	0.01
Kawaguchi et al (2016) [10]	Katana Avencia	PA 40%	3 months water	Clearfil Cer. Primer Plus	Panavia v5	µTBS	69.60	3	13.90	70.00	3	13.90	13.90	0.50	0.40	0.97	-31.11	31.91	0.03	0.02	0.82	-1.58	1.62	0.03
Kawaguchi et al (2016) [10]	Katana Avencia	Ultrass. cleaning	6 months water	Clearfil Cer. Primer Plus	Panavia v5	µTBS	64.60	3	9.00	70.00	3	13.90	11.71	0.30	5.40	0.60	-21.14	31.94	0.46	0.37	0.82	-1.25	1.98	0.39
Kawaguchi et al (2016) [10]	Katana Avencia	PA 40%	1 month water	Clearfil Cer. Primer Plus	Panavia v5	µTBS	70.10	3	11.60	70.00	3	13.90	12.80	0.41	0.10	0.99	-28.92	29.12	0.01	0.01	0.82	-1.59	1.61	0.01
Kawaguchi et al (2016) [10]	Katana Avencia	Ultrass. cleaning + PA 40%	1 month water	Clearfil Cer. Primer Plus	Panavia v5	µTBS	70.00	3	9.80	70.00	3	13.90	12.03	0.33	0.00	1.00	-27.26	27.26	0.00	0.00	0.82	-1.60	1.60	0.00
Kawaguchi et al (2016) [10]	Katana Avencia	Ultrass. cleaning	3 months water	Clearfil Cer. Primer Plus	Panavia v5	µTBS	72.70	3	10.40	70.00	3	13.90	12.28	0.36	2.70	0.80	-25.13	30.53	0.22	0.18	0.82	-1.43	1.78	0.19
Kawaguchi et al (2016) [10]	Katana Avencia	UC + AC	No water	Clearfil Cer. Primer Plus	Panavia v5	µTBS	82.90	3	11.70	70.00	3	13.90	12.85	0.41	12.90	0.29	-16.22	42.02	1.00	0.80	0.85	-0.86	2.46	0.93
Kawaguchi et al (2016) [10]	Katana Avencia	Ultrass. cleaning	1 month water	Clearfil Cer. Primer Plus	Panavia v5	µTBS	60.00	3	9.30	70.00	3	13.90	11.83	0.31	10.00	0.36	-16.81	36.81	0.85	0.67	0.84	-0.97	2.32	0.72
Kawaguchi et al (2016) [10]	Katana Avencia	Ultrass. cleaning	No water	Clearfil Cer. Primer Plus	Panavia v5	µTBS	87.60	3	8.90	70.00	3	13.90	11.67	0.29	17.60	0.14	-8.86	44.06	1.51	1.20	0.89	-0.54	2.94	1.27
Kawaguchi et al (2016) [10]	Katana Avencia	No cleaning treatment	No water	Clearfil Cer. Primer Plus	Panavia v5	µTBS	96.50	3	10.30	70.00	3	13.90	12.23	0.35	26.50	0.06	-1.23	54.23	2.17	1.73	0.96	-0.15	3.60	1.91
Kawaguchi et al (2016) [10]	Katana Avencia	No cleaning treatment	6 months water	Clearfil Cer. Primer Plus	Panavia v5	µTBS	78.00	3	14.40	70.00	3	13.90	14.15	0.48	8.00	0.53	-24.08	40.08	0.57	0.45	0.83	-1.17	2.07	0.58
Kawaguchi et al (2016) [10]	Katana Avencia	No cleaning treatment	3 months water	Clearfil Cer. Primer Plus	Panavia v5	µTBS	89.70	3	11.80	70.00	3	13.90	12.89	0.42	19.70	0.13	-9.53	48.93	1.53	1.22	0.89	-0.52	2.96	1.42
Liebermann et al (2013) [2]	ArtBlock temp	Al2O3 50 µm	24 h water + 5000 cycles	Visiolink	Clearfil SA	TBS	17.30	20	5.30	7.10	20	7.80	6.67	0.05	10.20	0.00	5.93	14.47	1.53	1.50	0.36	0.80	2.20	1.31
Liebermann et al (2013) [2]	ArtBlock temp	Al2O3 50 µm	24 h water + 5000 cycles	VP Connect	Clearfil SA	TBS	17.90	20	4.60	7.10	20	7.80	6.40	0.01	10.80	0.00	6.70	14.90	1.69	1.65	0.37	0.94	2.37	1.38
Liebermann et al (2013) [2]	ArtBlock temp	Al2O3 50 µm	24 h water + 5000 cycles	Visiolink	Clearfil SA	TBS	28.80	20	6.30	7.10	20	7.80	7.09	0.18	21.70	0.00	17.16	26.24	3.06	3.00	0.46	2.10	3.90	2.78
Liebermann et al (2013) [2]	ArtBlock temp	Al2O3 50 µm	24 h water + 5000 cycles	VP Connect	Clearfil SA	TBS	25.60	20	4.30	7.10	20	7.80	6.30	0.01	18.50	0.00	14.47	22.53	2.94	2.88	0.45	1.99	3.76	2.37
Liebermann et al (2013) [2]	ArtBlock temp	Al2O3 50 µm	24 h water + 5000 cycles	Visiolink	Rely X Unicem	TBS	29.00	20	6.30	2.00	20	6.20	6.25	0.47	27.00	0.00	23.00	31.00	4.32	4.23	0.57	3.12	5.35	4.35
Liebermann et al (2013) [2]	ArtBlock temp	Al2O3 50 µm	24 h water + 5000 cycles	NO	Rely X Unicem	TBS	10.20	20	11.90	2.00	20	6.20	9.49	0.00	8.20	0.01	2.13	14.27	0.86	0.85	0.33	0.20	1.49	1.32
Liebermann et al (2013) [2]	ArtBlock temp	Al2O3 50 µm	24 h water + 5000 cycles	Visiolink	Rely X Unicem	TBS	17.60	20	4.10	2.00	20	6.20	5.26	0.04	15.60	0.00	12.24	18.96	2.97	2.91	0.45	2.02	3.80	2.52
Liebermann et al (2013) [2]	ArtBlock temp	Al2O3 50 µm	24 h water + 5000 cycles	VP Connect	Rely X Unicem	TBS	2.10	20	4.10	2.00	20	6.20	5.26	0.04	0.10	0.95	-3.26	3.46	0.02	0.02	0.32	-0.60	0.64	0.02
Malysa et al (2022) [37]	IPS Empress CAD	HF 5%	No Thermal Cycling	NO	Maxcem	SBS	15.48	12	1.16	2.06	12	0.45	0.88	0.00	13.42	0.00	12.68	14.16	15.30	14.77	2.17	10.52	19.03	30.16

Malysa et al (2022) [37]	IPS Empress CAD	HF 5%	Thermal Cycling	NO	Maxcem	SBS	3.37	12	0.23	2.06	12	0.45	0.35	0.02	1.31	0.00	1.01	1.61	3.71	3.58	0.66	2.29	4.87	2.95
Malysa et al (2022) [37]	IPS Empress CAD	HF 5%	.	NO	Maxcem	SBS	12.11	12	1.27	2.06	12	0.45	0.95	0.00	10.05	0.00	9.24	10.86	10.53	10.17	1.52	7.18	13.16	22.59
Malysa et al (2022) [37]	IPS e max. Zircad	HF 5%	Thermal Cycling	NO	Maxcem	SBS	2.32	12	0.18	2.06	12	0.45	0.34	0.00	0.26	0.07	-0.03	0.55	0.77	0.74	0.42	-0.08	1.57	0.59
Malysa et al (2022) [37]	IPS e max. Zircad	HF 5%	No Thermal Cycling	NO	Maxcem	SBS	13.29	12	1.19	2.06	12	0.45	0.90	0.00	11.23	0.00	10.47	11.99	12.55	12.12	1.80	8.60	15.64	25.24
Malysa et al (2022) [37]	IPS e max. Zircad	HF 5%	.	NO	Maxcem	SBS	10.97	12	1.21	2.06	12	0.45	0.91	0.00	8.91	0.00	8.14	9.68	9.76	9.42	1.42	6.64	12.21	20.03
Malysa et al (2022) [37]	IPS e.max CAD	HF 5% + PA 37%	No Thermal Cycling	NO	Maxcem	SBS	8.43	12	0.87	2.06	12	0.45	0.69	0.02	6.37	0.00	5.79	6.95	9.24	8.93	1.35	6.28	11.57	14.31
Malysa et al (2022) [37]	IPS e.max CAD	HF 5% + PA 37%	.	NO	Maxcem	SBS	6.37	12	0.74	2.06	12	0.45	0.61	0.05	4.31	0.00	3.80	4.83	7.07	6.83	1.07	4.74	8.92	9.69
Malysa et al (2022) [37]	IPS Empress CAD	HF 5%	.	NO	Panavia SA	SBS	13.96	12	1.92	2.55	12	0.55	1.41	0.00	11.41	0.00	10.21	12.61	8.06	7.79	1.20	5.44	10.13	20.75
Malysa et al (2022) [37]	IPS Empress CAD	HF 5%	Thermal Cycling	NO	Panavia SA	SBS	2.98	12	0.67	2.55	12	0.55	0.61	0.27	0.43	0.10	-0.09	0.95	0.70	0.68	0.42	-0.14	1.50	0.78
Malysa et al (2022) [37]	IPS Empress CAD	HF 5%	No Thermal cycling	NO	Panavia SA	SBS	16.94	12	1.53	2.55	12	0.55	1.15	0.00	14.39	0.00	13.41	15.37	12.50	12.06	1.79	8.56	15.57	26.16
Malysa et al (2022) [37]	IPS e max. Zircad	HF 5%	.	NO	Panavia SA	SBS	15.77	12	1.42	2.55	12	0.55	1.08	0.00	13.22	0.00	12.31	14.13	12.25	11.82	1.75	8.39	15.26	24.04
Malysa et al (2022) [37]	IPS e max. Zircad	HF 5%	No Thermal cycling	NO	Panavia SA	SBS	18.31	12	1.36	2.55	12	0.55	1.04	0.00	15.76	0.00	14.88	16.64	15.22	14.70	2.16	10.46	18.93	28.65
Malysa et al (2022) [37]	IPS e.max CAD	HF 5% + PA 37%	.	NO	Panavia SA	SBS	6.68	12	1.16	2.55	12	0.55	0.91	0.01	4.13	0.00	3.36	4.90	4.54	4.39	0.75	2.91	5.86	7.51
Malysa et al (2022) [37]	IPS e.max CAD	HF 5% + PA 37%	No Thermal cycling	NO	Panavia SA	SBS	9.89	12	0.93	2.55	12	0.55	0.76	0.05	7.34	0.00	6.69	7.98	9.64	9.30	1.40	6.55	12.05	13.34
Malysa et al (2022) [37]	IPS e.max CAD	HF 5% + PA 37%	Thermal Cycling	NO	Panavia SA	SBS	3.21	12	0.59	2.55	12	0.55	0.57	0.41	0.66	0.01	0.18	1.14	1.16	1.12	0.44	0.26	1.98	1.20
Malysa et al (2022) [37]	IPS Empress CAD	HF 5%	Thermal Cycling	NO	Panavia V5	SBS	16.84	12	0.84	3.06	12	0.95	0.90	0.35	13.78	0.00	13.02	14.54	15.32	14.79	2.17	10.53	19.05	14.48
Malysa et al (2022) [37]	IPS Empress CAD	HF 5%	No Thermal cycling	NO	Panavia V5	SBS	20.33	12	0.79	3.06	12	0.95	0.87	0.27	17.27	0.00	16.53	18.01	19.77	19.09	2.79	13.63	24.55	18.14
Malysa et al (2022) [37]	IPS Empress CAD	HF 5%	.	NO	Panavia V5	SBS	3.49	12	1.04	3.06	12	0.95	1.00	0.39	0.43	0.30	-0.42	1.27	0.43	0.42	0.41	-0.39	1.22	0.45
Malysa et al (2022) [37]	IPS e max. Zircad	HF 5%	.	NO	Panavia V5	SBS	3.63	12	0.88	3.06	12	0.95	0.91	0.39	0.57	0.14	-0.20	1.35	0.63	0.61	0.42	-0.21	1.42	0.60
Malysa et al (2022) [37]	IPS e max. Zircad	HF 5%	Thermal Cycling	NO	Panavia V5	SBS	7.60	12	0.77	3.06	12	0.95	0.86	0.24	4.54	0.00	3.81	5.27	5.26	5.08	0.84	3.43	6.72	4.77
Malysa et al (2022) [37]	IPS e max. Zircad	HF 5%	No Thermal cycling	NO	Panavia V5	SBS	11.23	12	0.48	3.06	12	0.95	0.75	0.01	8.17	0.00	7.53	8.81	10.86	10.49	1.57	7.41	13.56	8.58
Malysa et al (2022) [37]	IPS e.max CAD	HF 5% + PA 37%	No Thermal cycling	NO	Panavia V5	SBS	22.50	12	0.69	3.06	12	0.95	0.83	0.15	19.44	0.00	18.74	20.14	23.38	22.58	3.28	16.14	29.01	20.42
Malysa et al (2022) [37]	IPS e.max CAD	HF 5% + PA 37%	Thermal Cycling	NO	Panavia V5	SBS	19.45	12	0.56	3.06	12	0.95	0.78	0.05	16.39	0.00	15.73	17.05	20.95	20.23	2.95	14.45	26.00	17.22
Malysa et al (2022) [37]	IPS Empress CAD	HF 5%	.	NO	Rely X U200	SBS	11.77	12	1.90	1.95	12	0.52	1.39	0.00	9.82	0.00	8.64	11.00	7.07	6.82	1.07	4.73	8.91	18.88
Malysa et al (2022) [37]	IPS Empress CAD	HF 5%	No Thermal cycling	NO	Rely X U200	SBS	15.14	12	1.95	1.95	12	0.52	1.43	0.00	13.19	0.00	11.98	14.40	9.23	8.91	1.35	6.27	11.56	25.37
Malysa et al (2022) [37]	IPS Empress CAD	HF 5%	Thermal Cycling	NO	Rely X U200	SBS	3.36	12	0.26	1.95	12	0.52	0.41	0.02	1.41	0.00	1.06	1.76	3.43	3.31	0.63	2.08	4.54	2.71
Malysa et al (2022) [37]	IPS e max. Zircad	HF 5% + PA 37%	No Thermal cycling	NO	Rely X U200	SBS	22.73	12	1.15	1.95	12	0.52	0.89	0.01	20.78	0.00	20.03	21.53	23.32	22.51	3.28	16.09	28.93	39.96
Malysa et al (2022) [37]	IPS e max. Zircad	HF 5%	.	NO	Rely X U200	SBS	20.78	12	1.20	1.95	12	0.52	0.92	0.01	18.83	0.00	18.05	19.61	20.42	19.71	2.87	14.08	25.35	36.21
Malysa et al (2022) [37]	IPS e.max CAD	HF 5% + PA 37%	.	NO	Rely X U200	SBS	5.09	12	0.91	1.95	12	0.52	0.74	0.04	3.14	0.00	2.52	3.77	4.25	4.10	0.72	2.69	5.51	6.04
Malysa et al (2022) [37]	IPS e.max CAD	HF 5% + PA 37%	Thermal Cycling	NO	Rely X U200	SBS	3.14	12	0.60	1.95	12	0.52	0.56	0.33	1.19	0.00	0.71	1.66	2.12	2.05	0.50	1.06	3.04	2.28
Malysa et al (2022) [37]	IPS e.max CAD	HF 5% + PA 37%	No Thermal cycling	NO	Rely X U200	SBS	8.23	12	0.70	1.95	12	0.52	0.61	0.17	6.28	0.00	5.76	6.80	10.22	9.87	1.48	6.96	12.77	12.07
Oda et al (2021) [25]	Katana Avencia	Al_2O_3 50 µm	24 hours water	Clearfil Cer. Primer Plus	Panavia SA Plus	µTBS	37.20	5	8.30	14.70	5	5.70	7.12	0.24	22.50	0.00	12.12	32.88	3.16	2.85	0.90	1.09	4.61	3.95
Oda et al (2021) [25]	Katana Avencia	Al_2O_3 50 µm	24 hours water	Clearfil Cer. Primer Plus	Panavia SA Plus	µTBS	17.00	5	3.70	14.70	5	5.70	4.81	0.21	2.30	0.47	-4.71	9.31	0.48	0.43	0.64	-0.82	1.69	0.40
Oda et al (2021) [25]	Katana Avencia	Al_2O_3 50 µm	24 hours water	Clearfil Cer. Primer Plus	Panavia SA Plus	µTBS	40.30	5	9.80	14.70	5	5.70	8.02	0.16	25.60	0.00	13.91	37.29	3.19	2.88	0.90	1.11	4.65	4.49
Oda et al (2021) [25]	Katana Avencia	Al_2O_3 50 µm	24 hours water	Clearfil Cer. Primer Plus	Panavia SA Plus	µTBS	23.90	5	6.80	14.70	5	5.70	6.27	0.37	9.20	0.05	0.05	18.35	1.47	1.32	0.70	-0.04	2.69	1.61
Oda et al (2021) [25]	Katana Avencia	Al_2O_3 50 µm	24 hours water	Clearfil Cer. Primer Plus	Panavia SA Univ.	µTBS	24.50	5	6.20	14.70	5	5.70	5.96	0.44	9.80	0.03	1.11	18.49	1.65	1.49	0.71	0.09	2.89	1.72
Oda et al (2021) [25]	Katana Avencia	Al_2O_3 50 µm	24 hours water	Clearfil Cer. Primer Plus	Panavia SA Univ.	µTBS	34.20	5	8.10	14.70	5	5.70	7.00	0.26	19.50	0.00	9.29	29.71	2.78	2.51	0.85	0.86	4.17	3.42
Oda et al (2021) [25]	Katana Avencia	Al_2O_3 50 µm	24 hours water	Clearfil Cer. Primer Plus	Panavia SA Univ.	µTBS	29.50	5	7.00	14.70	5	5.70	6.38	0.35	14.80	0.01	5.49	24.11	2.32	2.09	0.79	0.55	3.64	2.60
Poggio et al (2016) [6]	Lava Ultimate	No treatment	24h water	SB-UA	Rely X Ultimate	SBS	16.58	10	3.87	11.98	10	2.91	3.42	0.20	4.60	0.01	1.38	7.82	1.34	1.29	0.49	0.32	2.25	1.58

Takahashi et al (2022) [42]	Estelite block	Al2O3 50 μm	Thermocycling 30 000 cycles	HC Primer	Block HC Cem	SBS	31.30	10	3.60	21.60	10	2.50	3.10	0.15	9.70	0.00	6.79	12.61	3.13	3.00	0.65	1.72	4.27	3.88
Takahashi et al (2022) [42]	Estelite block	Al2O3 50 μm	Thermocycling 10 000 cycles	HC Primer	Block HC Cem	SBS	34.80	10	3.30	21.60	10	2.50	2.93	0.21	13.20	0.00	10.45	15.95	4.51	4.32	0.82	2.72	5.92	5.28
Takahashi et al (2022) [42]	Estelite block	Al2O3 50 μm	24h water	HC Primer	Block HC Cem	SBS	24.10	10	2.30	21.60	10	2.50	2.40	0.40	2.50	0.03	0.24	4.76	1.04	1.00	0.47	0.07	1.93	1.00
Takahashi et al (2022) [42]	Estelite block	Al2O3 50 μm	15 min water	HC Primer	Block HC Cem	SBS	24.50	10	1.00	21.60	10	2.50	1.90	0.01	2.90	0.00	1.11	4.69	1.52	1.46	0.50	0.47	2.44	1.16
Takahashi et al (2022) [42]	Katana Avencia	Al2O3 50 μm	Thermocycling 10 000 cycles	HC Primer	Block HC Cem	SBS	23.50	10	2.90	21.60	10	2.50	2.71	0.33	1.90	0.13	-0.64	4.44	0.70	0.67	0.46	-0.23	1.57	0.76
Takahashi et al (2022) [42]	Katana Avencia	Al2O3 50 μm	24h water	HC Primer	Block HC Cem	SBS	24.60	10	3.00	21.60	10	2.50	2.76	0.30	3.00	0.03	0.41	5.59	1.09	1.04	0.48	0.11	1.97	1.20
Takahashi et al (2022) [42]	Katana Avencia	Al2O3 50 μm	15 min water	HC Primer	Block HC Cem	SBS	24.30	10	3.00	21.60	10	2.50	2.76	0.30	2.70	0.04	0.11	5.29	0.98	0.94	0.47	0.01	1.86	1.08
Takahashi et al (2022) [42]	Shofu Block HC	Al2O3 50 μm	15 min water	HC Primer	Block HC Cem	SBS	25.50	10	2.50	21.60	10	2.50	2.50	0.50	3.90	0.00	1.55	6.25	1.56	1.49	0.51	0.50	2.49	1.56
Takahashi et al (2022) [42]	Shofu Block HC	Al2O3 50 μm	24h water	HC Primer	Block HC Cem	SBS	29.30	10	2.50	21.60	10	2.50	2.50	0.50	7.70	0.00	5.35	10.05	3.08	2.95	0.65	1.68	4.22	3.08
Takahashi et al (2022) [42]	Shofu Block HC	Al2O3 50 μm	Thermocycling 30 000 cycles	HC Primer	Block HC Cem	SBS	19.70	10	2.70	21.60	10	2.50	2.60	0.41	1.90	0.12	-0.54	4.34	0.73	0.70	0.46	-0.20	1.60	0.76
Takahashi et al (2022) [42]	Shofu Block HC	Al2O3 50 μm	Thermocycling 10 000 cycles	HC Primer	Block HC Cem	SBS	26.50	10	3.50	21.60	10	2.50	3.04	0.17	4.90	0.00	2.04	7.76	1.61	1.54	0.51	0.54	2.54	1.96
Takahashi et al (2022) [42]	Estelite block	Al2O3 50 μm	Thermocycling 30 000 cycles	HC Primer	Panavia SA Univ.	SBS	14.70	10	3.00	12.10	10	1.20	2.28	0.01	2.60	0.02	0.45	4.75	1.14	1.09	0.48	0.15	2.03	2.17
Takahashi et al (2022) [42]	Estelite block	Al2O3 50 μm	Thermocycling 10 000 cycles	HC Primer	Panavia SA Univ.	SBS	17.70	10	3.40	12.10	10	1.20	2.55	0.00	5.60	0.00	3.20	8.00	2.20	2.10	0.56	1.01	3.20	4.67
Takahashi et al (2022) [42]	Estelite block	Al2O3 50 μm	15 min water	HC Primer	Panavia SA Univ.	SBS	12.10	10	1.20	12.10	10	1.20	1.20	0.50	0.00	1.00	-1.13	1.13	0.00	0.00	0.45	-0.88	0.88	0.00
Takahashi et al (2022) [42]	Estelite block	Al2O3 50 μm	24h water	HC Primer	Panavia SA Univ.	SBS	17.90	10	3.40	12.10	10	1.20	2.55	0.00	5.80	0.00	3.40	8.20	2.27	2.18	0.56	1.07	3.29	4.83
Takahashi et al (2022) [42]	Katana Avencia	Al2O3 50 μm	Thermocycling 30 000 cycles	HC Primer	Panavia SA Univ.	SBS	18.60	10	2.20	12.10	10	1.20	1.77	0.04	6.50	0.00	4.84	8.16	3.67	3.51	0.71	2.12	4.91	5.42
Takahashi et al (2022) [42]	Katana Avencia	Al2O3 50 μm	24h water	HC Primer	Panavia SA Univ.	SBS	19.90	10	1.80	12.10	10	1.20	1.53	0.12	7.80	0.00	6.36	9.24	5.10	4.88	0.89	3.13	6.63	6.50
Takahashi et al (2022) [42]	Katana Avencia	Al2O3 50 μm	15 min water	HC Primer	Panavia SA Univ.	SBS	15.60	10	1.90	12.10	10	1.20	1.59	0.09	3.50	0.00	2.01	4.99	2.20	2.11	0.56	1.02	3.20	2.92
Takahashi et al (2022) [42]	Katana Avencia	Al2O3 50 μm	Thermocycling 10 000 cycles	HC Primer	Panavia SA Univ.	SBS	19.80	10	1.90	12.10	10	1.20	1.59	0.09	7.70	0.00	6.21	9.19	4.85	4.64	0.86	2.96	6.32	6.42
Takahashi et al (2022) [42]	Shofu Block HC	Al2O3 50 μm	15 min water	HC Primer	Panavia SA Univ.	SBS	14.50	10	1.50	12.10	10	1.20	1.36	0.26	2.40	0.00	1.12	3.68	1.77	1.69	0.52	0.67	2.71	2.00
Takahashi et al (2022) [42]	Shofu Block HC	Al2O3 50 μm	Thermocycling 10 000 cycles	HC Primer	Panavia SA Univ.	SBS	19.80	10	3.50	12.10	10	1.20	2.62	0.00	7.70	0.00	5.24	10.16	2.94	2.82	0.63	1.58	4.06	6.42
Takahashi et al (2022) [42]	Shofu Block HC	Al2O3 50 μm	Thermocycling 30 000 cycles	HC Primer	Panavia SA Univ.	SBS	18.50	10	2.70	12.10	10	1.20	2.09	0.01	6.40	0.00	4.44	8.36	3.06	2.93	0.64	1.67	4.20	5.33
Takahashi et al (2022) [42]	Shofu Block HC	Al2O3 50 μm	24h water	HC Primer	Panavia SA Univ.	SBS	16.10	10	1.80	12.10	10	1.20	1.53	0.12	4.00	0.00	2.56	5.44	2.61	2.50	0.60	1.33	3.67	3.33
Ustun et al (2021) [27]	Cerasmart	HF 5%	Thermal aging	Ultradent Silane	Rely X U200	μSBS	5.62	7	0.29	4.69	7	0.21	0.25	0.23	0.93	0.00	0.64	1.22	3.67	3.45	0.84	1.80	5.11	4.43
Ustun et al (2021) [27]	Cerasmart	HF 5%	No Thermal aging	Ultradent Silane	Rely X U200	μSBS	7.48	7	0.32	4.69	7	0.21	0.27	0.16	2.79	0.00	2.47	3.11	10.31	9.69	1.91	5.95	13.43	13.29
Ustun et al (2021) [27]	Vita Enamic	HF 5%	No Thermal aging	Ultradent Silane	Rely X U200	μSBS	7.14	7	0.45	4.69	7	0.21	0.35	0.04	2.45	0.00	2.04	2.86	6.98	6.56	1.35	3.91	9.20	11.67
Ustun et al (2021) [27]	Vita suprinity	HF 5%	No Thermal aging	Ultradent Silane	Rely X U200	μSBS	10.27	7	0.54	4.69	7	0.21	0.41	0.02	5.58	0.00	5.10	6.06	13.62	12.80	2.48	7.95	17.66	26.57
Ustun et al (2021) [27]	Vita suprinity	HF 5%	Thermal aging	Ultradent Silane	Rely X U200	μSBS	8.46	7	0.33	4.69	7	0.21	0.28	0.15	3.77	0.00	3.45	4.09	13.63	12.81	2.48	7.95	17.67	17.95
Ustun et al (2021) [27]	Cerasmart	HF 5%	Thermal aging	Ultradent Silane	Rely X Ultimate	μSBS	7.57	7	0.37	4.59	7	0.41	0.39	0.40	2.98	0.00	2.53	3.43	7.63	7.17	1.46	4.32	10.03	7.27
Ustun et al (2021) [27]	Cerasmart	HF 5%	No Thermal aging	Ultradent Silane	Rely X Ultimate	μSBS	9.89	7	0.32	4.59	7	0.41	0.37	0.28	5.30	0.00	4.87	5.73	14.41	13.55	2.62	8.42	18.67	12.93
Ustun et al (2021) [27]	Cerasmart	PA 37%	No Thermal aging	Ultradent Silane	Rely X Ultimate	μSBS	8.05	7	0.26	4.59	7	0.41	0.34	0.15	3.46	0.00	3.06	3.86	10.08	9.47	1.87	5.81	13.14	8.44
Ustun et al (2021) [27]	Cerasmart	PA 37%	Thermal aging	Ultradent Silane	Rely X Ultimate	μSBS	6.29	7	0.33	4.59	7	0.41	0.37	0.31	1.70	0.00	1.27	2.13	4.57	4.29	0.97	2.39	6.20	4.15
Ustun et al (2021) [27]	Vita Enamic	PA 37%	No Thermal aging	Ultradent Silane	Rely X Ultimate	μSBS	7.14	7	0.12	4.59	7	0.41	0.30	0.00	2.55	0.00	2.20	2.90	8.44	7.94	1.59	4.81	11.06	6.22
Ustun et al (2021) [27]	Vita Enamic	HF 5%	Thermal aging	Ultradent Silane	Rely X Ultimate	μSBS	6.37	7	0.33	4.59	7	0.41	0.37	0.31	1.78	0.00	1.35	2.21	4.78	4.50	1.00	2.53	6.46	4.34
Ustun et al (2021) [27]	Vita Enamic	HF 5%	No Thermal aging	Ultradent Silane	Rely X Ultimate	μSBS	8.54	7	0.31	4.59	7	0.41	0.36	0.26	3.95	0.00	3.53	4.37	10.87	10.22	2.00	6.29	14.14	9.63
Ustun et al (2021) [27]	Vita suprinity	PA 37%	Thermal aging	SB-UA	Rely X Ultimate	μSBS	8.34	7	0.30	4.59	7	0.41	0.36	0.23	3.75	0.00	3.33	4.17	10.44	9.81	1.93	6.03	13.60	9.15
Ustun et al (2021) [27]	Vita suprinity	PA 37%	No Thermal aging	Ultradent Silane	Rely X Ultimate	μSBS	10.90	7	0.34	4.59	7	0.41	0.38	0.33	6.31	0.00	5.87	6.75	16.75	15.75	3.02	9.82	21.68	15.39
Ustun et al (2021) [27]	Vita suprinity	HF 5%	Thermal aging	Ultradent Silane	Rely X Ultimate	μSBS	5.48	7	0.35	4.59	7	0.41	0.38	0.36	0.89	0.00	0.45	1.33	2.33	2.19	0.68	0.87	3.52	2.17
Ustun et al (2021) [27]	Vita suprinity	HF 5%	No Thermal aging	Ultradent Silane	Rely X Ultimate	μSBS	7.47	7	0.46	4.59	7	0.41	0.44	0.39	2.88	0.00	2.37	3.39	6.61	6.21	1.29	3.68	8.74	7.02
